# Optimizing irrigation and nitrogen fertilization for seed yield in western wheatgrass [*Pascopyrum smithii* (Rydb.) Á. Löve] using a large multi-factorial field design

**DOI:** 10.1371/journal.pone.0218599

**Published:** 2019-06-26

**Authors:** Zhao Chen, Xv Liu, Junpeng Niu, Wennan Zhou, Tian Zhao, Wenbo Jiang, Jian Cui, Robert Kallenbach, Quanzhen Wang

**Affiliations:** 1 Department of Grassland Science, College of Grassland Agriculture, Northwest A&F University, Yangling, Shaanxi Province, China; 2 Department of Plant Science, College of Life Science, Northwest A&F University, Yangling, Shaanxi Province, China; 3 Division of Plant Sciences, University of Missouri, Columbia, MO, United States of America; Harran University, Sanliurfa, TURKEY

## Abstract

It is crucial for agricultural production to identify the trigger that switches plants from vegetative to reproductive growth. Agricultural sustainability in semiarid regions is challenged by nitrogen (N) fertilizer overuse, inadequate soil water, and heavy carbon emissions. Previous studies focused on the short-term effects of a single application of N and water but have not investigated the long-term effects of different irrigation and N fertilizer regimens on crop yields and yield components. N application is routinely coupled with water availability, and crop yields can be maximized by optimizing both. We examined the growth of western wheatgrass [*Pascopyrum smithii* (Rydb.) Á. Löve], a temperate-region forage and turf grass, using multiple different combinations of N fertilizer [(NH_4_)_2_·CO_3_] and irrigation levels over 3 years to determine optimal field management. We conducted multifactorial, orthogonally designed field experiments with large sample sizes, and measured fertile tillers m^-2^ (Y_1_), spikelets/fertile tillers (Y_2_), florets/spikelet (Y_3_), seed numbers/spikelet (Y_4_), seed weight (Y_5_), and seed yield (Z) to study factors associated with the switch between vegetative and reproductive growth. Fertilization had a greater effect on seed yield and yield components than irrigation. Y_1_ had the strongest positive effect on Z, whereas Y_5_ had a negative effect on Z. Irrigation and fertilization affected Z, Y_1_, and Y_5_. Fertilizer concentrations were positively correlated with Z, Y_1_, and Y_5_, whereas irrigation levels were negatively correlated. The ridge regression linear model results suggested N application rate and irrigation had antagonistic effects on Y_1_ (X_3_ = 867.6–4.23×X_2_; R^2^ = 0.988, F = Infinity, *P*<0.0001). We conclude that the optimal amount of N fertilizer and irrigation was 156 kg ha^-1^ + 115 mm for seed yield, 120 kg ha^-1^ + 146 mm for spikelets/fertile tillers, and 108 kg ha^-1^ + 119 mm for seed numbers/spikelets. These results will improve yield and reduce agricultural inputs for *P*. *smithii* in semiarid and arid regions, thereby reducing fertilizer pollution and conserving water.

## Introduction

Large volumes of irrigation water are required to ensure high grain yields in northwest China and other arid regions [1, 2]. Accelerated industrialization and urbanization have increased water demands and competition for water among agricultural functions, industries, and households [3, 4]. Excessive fertilizer use in modern agriculture has caused environmental pollution and increased water demands. Nitrogen (N) fertilizer [5] causes the largest environmentally significant losses from N leaching and N_2_O emissions [6]. Thus, we urgently need to increase grain yields using less water and fertilizer by developing water-saving and N-efficient protocols for field management and environmentally responsible seed production.

Irrigation and N management are crucial for maintaining grass growth in arid areas, and it is vital to optimize these linked factors for sustainable agricultural management [7]. Water deficiency results in high fertilizer expenses [8–10], and excessive fertilizer application has become a serious concern for the sustainable development of crop seed production [11]. Many studies report that the interaction between N supply and irrigation management affects N absorption/utilization and tomato and rice yields [12, 13]. For example, the observed interactions between N application and irrigation in maize, potato, and rice result in an optimal rate of N application for different water levels [8, 14, 15]. Appropriate irrigation schedules can reduce N loss, enhance crop growth, and increase yields [7, 16]. These results highlight the complex effects of water and N on vegetative and reproductive growth [17, 18]. However, there are few comprehensive field studies that evaluate the effects of different N fertilizer and irrigation regimens on seed production.

Western wheatgrass [*Pascopyrum smithii* (Rydb.) Á. Löve] is a perennial cool-season grass native to the southern mixed-grass prairie region of the Great Plains [19], and it is a rich genetic resource [20]. It is competitive, high-yielding, and provides an excellent forage for animal husbandry; it also significantly enhances soil protection and water conservation in temperate regions [21]. However, the seed yields of cool-season perennial grasses are often low, perhaps because of insufficient nutrients to adequately supply developing florets [22]. In China, the supply of perennial grass seeds depends on imports due to inadequate supplies of locally produced high-quality seed. The Chinese government encouraged the development of increased grass seed production capacity to enable greater self-sufficiency [23]. Perennial grass seed yields are affected by several factors [24]. Seed yield is positively correlated with plant height, ear diameter, number of seeds per row, and number of rows per ear [25]. An early study showed that seed yield is correlated with the number of grains per row, number of rows per ear, and 1000-grain weight [26]. The grain yield per plant is positively correlated with the 1000-grain weight, number of kernels per ear, ear weight, and ear insertion height [16]. To improve seed yield, we must manage N fertilizer and irrigation regimens and carefully observe the relationships between seed yield and plant reproductive traits. For example, seed yields in many grass species depend on the following reproductive factors: pods per plant, number of seeds per pod, number of fertile spikelets per panicle, panicle length, spikelet density, number of filled seeds, number of effect tillers per plant, and 1000 seed weight [27, 28].

Crop simulation models are often constrained by limitations in field data. Orthogonal experimental design (OED) is used to study the comparative effectiveness of multiple interventions and simultaneously determine optimum factor combinations with fewer experimental units than would be required to test all possible intervention combinations [29]. OED with orthogonal array and factor analysis enables optimization of factor combinations with fewer tests [29]. Seed yield is affected by crop species, environmental conditions, and agronomic factors [30]. OED enables simultaneous validation of numerous interventions in a unitary test and possible identification of some interactions [29]. An accurate method is needed to estimate how irrigation and fertilization management practices influence seed yields and related factors [31]. Therefore, we conducted a three-year field experiment that included multiple fertilization strategies and irrigation regimens. There may be synergistic or antagonistic interactions between fertilization and irrigation regimens, and these interactions may increase crop yield and related factors [32]. This study had three primary objectives. (1) Quantify the direct and indirect effects of N fertilizer and irrigation on seed yield and other yield factors in western wheatgrass. (2) Optimize N fertilizer and irrigation to obtain the highest seed yield and enhance seed yield factors. (3) Identify the synergistic or antagonistic interactions between N fertilizer and irrigation, and investigate the relationship(s) between vegetative and reproductive growth in western wheatgrass.

## Methods

### Experimental site

The *P*. *smithii* cultivar was introduced into China from the United States in 2002, according to the 948 project (202009) of the Ministry of Agriculture of China. Field experiments were conducted during three growing seasons (2003–2005) at the China Agricultural University Grassland Research Station located in the Hexi Corridor in Jiuquan, Gansu province, northwestern China (39°37′N, 98°30′E; 1,480 m above sea level). The site contains Mot-Cal-Orthic aridisols, which are classified as Xeric Haplocalcids by the United States Department of Agriculture soil classification guidelines [33]. The site is located inland, with high latitude and altitude, and involves a typical continental monsoon climate, which is dry with scarce precipitation, intense evaporation, and large temperature differences between day and night. Records from the Jiuquan Meteorological Station from 1995 to 2004 indicate that the annual average minimum and maximum air temperatures were –15.6°C and 28.7°C, respectively, and the annual average temperature was 7.3°C. The average air temperature and precipitation were measured at a weather station near the experimental site during the crop growing seasons of the three-year study (S1 Fig). The soil (0–20 cm) chemical characteristics were as follows: pH 8.39; 32.32 mg kg^-1^ NH_4_^+^; 20.09 mg kg^-1^ NO_3_; 118.30 mg kg^-1^ alkali hydrolysable nitrogen; 36.56 mg kg^-1^ available phosphorus; 130.30 mg kg^-1^ available potassium; 0.764 g kg^-1^ total nitrogen; 0.814 g kg^-1^ total phosphorus; 12.52g kg^-1^ total potassium; and 10.32 g kg^-1^ organic matter (S1 Table). The plots in this study had been planted with alfalfa (*Medicago sativa* L.) in the preceding season. The 6,000 m^2^ experimental site was tilled in the fall using a chisel plough, and a disk-harrow was used in the spring to prepare seedbeds. *P*. *smithii* seeds were planted on April 23, 2002 at a depth of 2.5 cm, the seeding rate was 5×10^6^ seeds ha^-1^, and the inter-row distance was 0.45 m. The initial fertilizer treatment was applied in a band that was 6 cm deep and 5 cm to the side of the seed furrows, at a rate of 104 kg ha^-1^ N and 63 kg ha^-1^ P_2_O_5_. There was no seed yield in autumn 2002. The plot was irrigated five times during the growth season, including the green period, jointing period, earing period, filled period, and flowering period, respectively. The plot was fertilized before sowing and returning to green in the next spring.

### Experimental design

We used six groups (A to F) to simulate different growing conditions (S2–S13 Tables). Each group tested two to six factors (X_1_ to X_6_). We were able to design experiments to identify the best combination of treatments with the fewest number of tests because the OED enables equilibrium dispersion. The OED also allowed us to transform complex multifactor data to single factor analysis. We used multifactorial orthogonal designs based on these six groups (S2 Table) [34–36] for a total of 380 experimental plots under various field treatments (S2 Table), each with an area of 28 m^2^ (i.e., 4 × 7 m) with 1.5 m spacing between adjacent plots in a 4100 m^2^ field. The treatments included 16 levels of P fertilizer (X_4_), 18 levels of N fertilizer (X_3_), 11 levels of surface drip irrigation (X_2_), planting densities (X_5_), and spray plant regulators (X_1_) (S4 Table). The levels of these factors were as follows. (1) The 11 levels of surface drip irrigation (X_2_) ranged from 0–148.1 mm yr^-1^: low water (LW) was 0–90.2 mm; middle water (MW) was 91–119.2 mm; and high water (HW) was 130–148.1 mm. (2) The 18 levels of N fertilizer (X_3_) ranged from 0–480 kg ha^-1^: low N (LN) was 0–100 kg ha^-1^ yr^-1^; middle N (MN) was 107–153 kg ha^-1^ yr^-1^; and high N (HN) was 176–480 kg ha^-1^ yr^-1^. (3) The 16 levels of P fertilizer (X_4_) as urea ranged from 0–240 kg ha^-1^: low P (LP) was 0–63 kg ha^-1^ yr^-1^; middle P (MP) was 77–105 kg ha^-1^ yr^-1^; and high P (HP) was 124–240 kg ha^-1^ yr^-1^. The planting densities (X_5_) and spray plant regulators (X_1_) also were varied (S4 Table) [37, 38]. Groups A and B each consisted of a two dimensional (2D) optimal design matrix arranged with different values for X_3_ and X_4_, respectively (S2 Table). Group C was organized according to a five-factor orthogonal design (X_1_ to X_5_). Groups D and E were organized as a two-factor orthogonal contrast plot (X_2_, X_3_+X_4_) and a three-factor orthogonal rotary design (X_1_, X_3_, and X_5_), respectively. We measured each of the following seed yield components for each plot during three successive years of the experiment: fertile tillers m^-2^ (Y_1_), spikelets/fertile tillers (Y_2_), florets/spikelet (Y_3_), seed numbers/spikelet (Y_4_), seed weight (Y_5_), and the resulting seed yield (Z). We also investigated and modeled the interactive effects of N application rate (X_3_) and irrigation (X_2_). The basic nutrient content of experimental soil is listed in S1 Table.

### Data collection

Ten samples along 1 m of each row were randomly selected to measure the five seed yield components from anthesis to seed harvest during 2003 to 2005. To avoid marginal utility, plants that were spaced 1 m or less from the plot edge were not sampled. Seed yield components and seed yield data for each plot were collected as follows: Y_1_ was measured from 10 randomly selected 1 m row samples; Y_2_, Y_3_, and Y_4_ were measured from 30 randomly selected fertile tillers and spikelets. The seed heads became ripe during the month of August from 2003 to 2005; we separately hand-threshed four samples from a 1-m length of the row, cleaned and weighed seeds from each sample, and determined that seed water content was 7–10% to convert weight values into kg ha^-1^ seed yield (Z) [39]. Ten lots of 100 seeds each were collected to determine the seed weight (Y_5_). The total number of samples (*n*) used to measure Y_1_–Y_5_ and Z were 3800, 13,605, 11,085, 10,770, 3800, and 1520, respectively, across all three years (S3 Table).

### Statistical analysis and analytical methods

We used path coefficient analysis to determine trait effects, both direct effects and indirect effects via other characters [40]. Knowledge of inter-character relationships is crucial in plant breeding for indirect selection of characters that are not easily measured and that have low heritability. Correlation coefficients can be used to predict changes in specific traits that are caused by selective pressures imposed on other traits. We performed correlation and path analyses to determine the relationships among yield and yield-contributing characters. Separate and combined analyses for the three study years provided useful information [41]. We analyzed data using ANOVA to determine the effects of irrigation, N fertilizer levels, and their interactions with Y_1_, Y_2_, Y_3_, Y_4_, Y_5_, and Z, according to a strip-plot design. We assessed significant differences between treatments using Tukey’s multiple range test (*P*<0.05). We wrote a Qbasic program for path coefficient analysis. All analyses were performed using SAS version 8.0. We also conducted simple linear and polynomial regression analyses to determine the relationships among applied N rates, irrigation rates, and the factors Y_1_–Y_5_ and Z, and determined the linear or quadratic nature of regression models based on the regression coefficients. Regression coefficients were significantly different from zero at *P* < 0.05. For the quadratic regression models, we calculated the optimal N and irrigation rates based on seed yield and yield components by setting the quadratic function to zero after the first derivative. Quadratic two-variable regression models comparing dependent variables (Y_1_–Y_5_ and Z, denoted as W) and independent variables (X_2_ and X_3_) were used as follows:

$$W=\sum_{i=1}^{2}\left(\beta_{i\times j+1}X_{j}^{i})+u\;(i=2,3;j=2,3\right)$$ (1)

where *β* is a constant.

**Fig 1:**
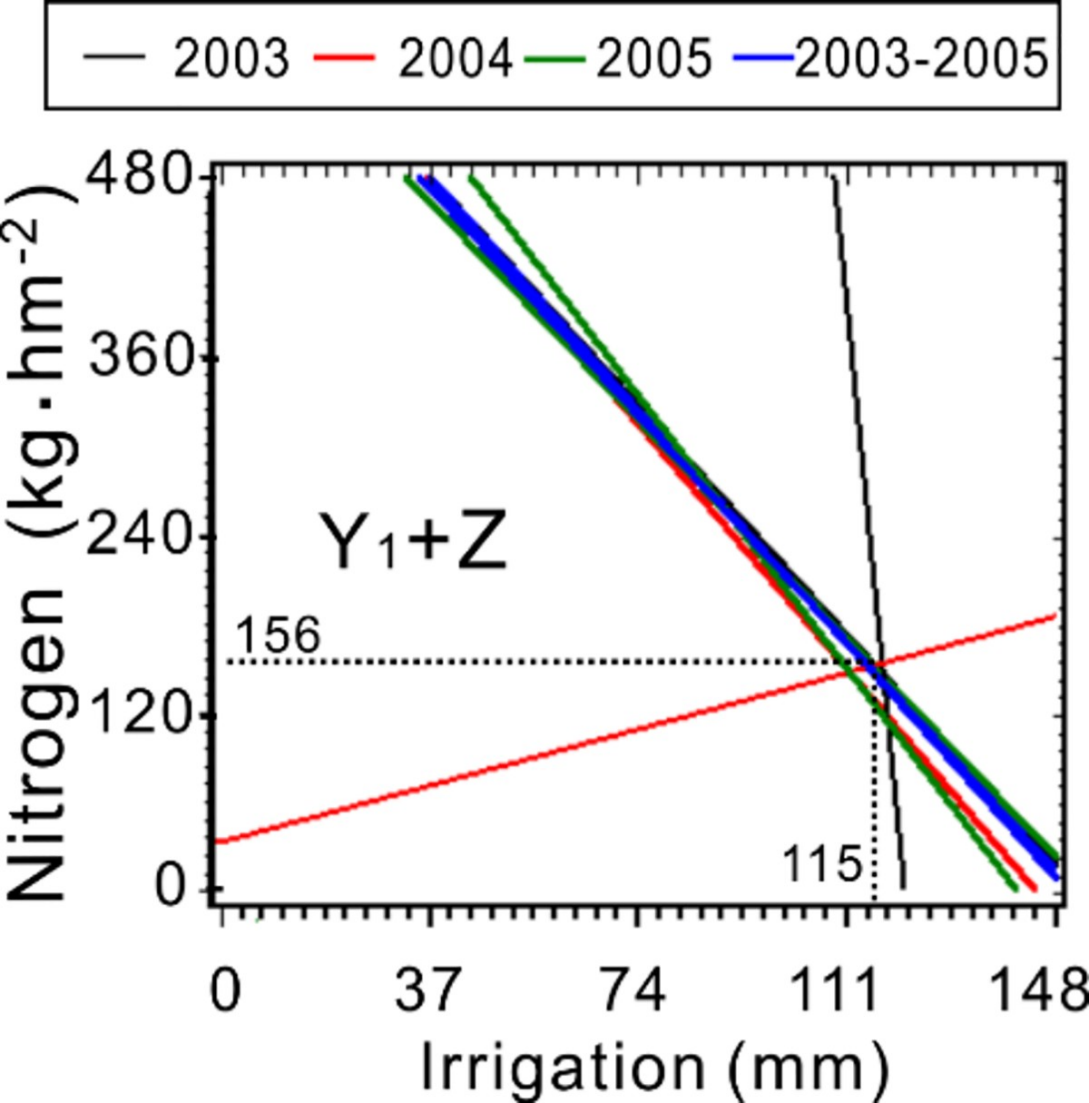
Ridgelines of the seed yield and seed yield component models.

**Fig 2:**
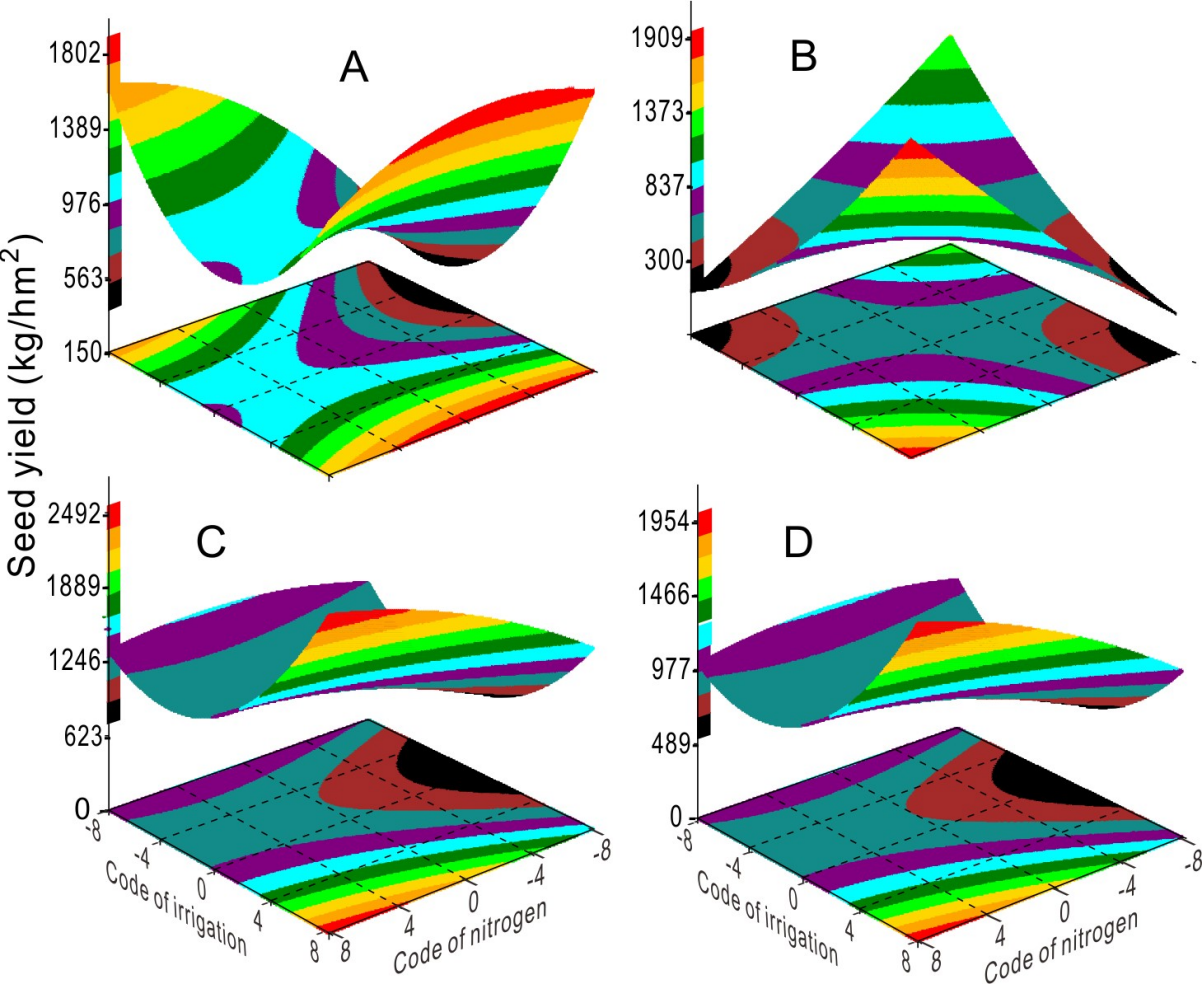
Response surface shows the coupling effects of irrigation and nitrogen fertilizer on seed yield (Z) for 2003 (A), 2004 (B), 2005 (C), and 2003–2005 (D).

The equivalent effects of X_i_ and X_j_ were determined as follows:

$$\frac{\partial W}{\partial X_{i}}=\frac{\partial W}{\partial X_{j}}$$ (2)

which produced the following ridgelines:

$$X_{3}=k\times X_{2}\pm b$$ (3)

where *b* is a constant. The ridgelines in Eq (3) (Fig 1) correspond to the response surface models [Eq (1)] to show the synergetic and antagonistic effects among traits [42]. We generated a response surface for Y_1_–Y_5_ and Z for their respective response variables (Fig 2). All analyses and graphing were performed with SAS version 8.2.

## Results

### Coupling the effects of irrigation and N fertilizer

N application and irrigation rates strongly affected seed yield (Z) in the three consecutive growing seasons. The F test values of Z in 2003, 2004, and 2005 were much larger for the N rate (133.819, 46.550, and 51.567, respectively) than for the irrigation rate (14.025, 15.154, and 15.397, respectively), and the water × N interaction was significant for Z (*P*<0.001) (Tables 1 to 3). The Pearson correlation analysis revealed that Z was closely related to fertile tillers (0.377, *P*<0.001 in 2003; 0.286, *P*<0.001 in 2004; 0.533, *P*<0.001 in 2005), spikelets/fertile tillers (0.129, *P*<0.001 in 2003; 0.119, *P*<0.001 in 2004), and seed weight (–0.220, *P*<0.001 in 2003; –0.229, *P*<0.001 in 2005), except in 2004 (Table 2). Seed yield was positively correlated with Y_1_ (correlation coefficient ranging from 0.125 to 0.533) and Y_2_ [correlation coefficients of 0.129 (2003), 0.119 (2004), and 0.172 (2003–2005)] among the five yield components. By contrast, Z was negatively affected by Y_4_ and Y_5_ during the experimental years (*P*≤0.05 or *P*≤0.01), and Z was not affected by Y_3_ during the experimental years. Y_1_ was significantly affected by irrigation and N application (*P*< 0.001 during 2003–2005; Table 3). The interaction effects of irrigation and N on the number of productive tillers was significant (*P*<0.001 during 2003–2005; Table 3). We observed the maximum number of productive tillers (1153) in 2003 (Table 1). The number of grains/spikelet (Y_2_) was significantly affected by irrigation in all comparisons (*P*<0.006), and significantly affected by N during 2003–2005 (*P*<0.001; Table 3). The maximum number of grains/spike (17.61 in 2003, 18.81 in 2004, and 18.76 in 2005) was observed in 2004 (Table 1). There was a significant interaction between irrigation and N on grains/spike (*P*<0.01) in 2003 and 2004 (Table 3). Y_1_ and Y_2_ showed strong intercorrelations, and Y_1_ was significantly negatively correlated with Y_3_ and Y_4_ in all three experimental years (*P*≤0.001; Table 2). The results indicated that Y_1_, Y_5_, and Z were significantly affected by irrigation (*P*<0.01) in all three experimental years (Tables 2 and 3), with variations in the response to irrigation at different water dosages. N fertilizer levels significantly affected seed yield and yield components in all three experimental years (*P*<0.01) except for Y_4_ (Tables 2 and 3). These results suggest that responses to changes in N supply differed depending on specific environmental conditions (other than the fixed variables in the study) during the experimental year. We also identified a significant interaction between N and irrigation for Y_1_, Y_5_, and Z in all experimental years (*P*<0.001), and for Y_2_ and Y_4_ in two experimental years (*P*<0.01; Table 3). We identified significant random effects from the year and relationships among seed yield components (Y_1_–Y_5_ and Z), which were detected using the PROC MIXED analysis of variance (ANOVA) in SAS (Table 4).

**Table 1:** Statistics of Y_1_–Y_5_ and Z in *Pascopyrum smithii* growing under six group experiments during 2003–2005.

Variable	Year	N	Mean	Std Dev	Std Error	Minimum	Maximum	CV	Pr>|t|
Y_1_	2003	105	674.4784	178.2814	17.3985	192.38	1153.33	0.27	<0.0001
	2004	129	267.7532	126.9550	1.9660	34	916	0.48	<0.0001
	2005	146	434.7001	161.2333	13.3438	167.17	830.67	0.37	<0.0001
Y_2_	2003	105	17.6083	0.9908	0.0967	15.21	20.21	0.06	<0.0001
	2004	129	18.8091	4.3803	0.0678	9	40	0.23	<0.0001
	2005	146	18.7603	1.3885	0.1149	15.63	22.03	0.08	<0.0001
Y_3_	2003	105	12.6991	1.6743	0.1634	8.84	17.12	0.13	<0.0001
	2004	129	7.5309	1.6297	0.0252	3	16	0.22	<0.0001
	2005	146	7.5742	0.5718	0.0473	6.4	9.03	0.08	<0.0001
Y_4_	2003	105	5.8180	0.4572	0.0446	4.39	6.64	0.08	<0.0001
	2004	129	4.8165	1.6596	0.0257	1	13	0.35	<0.0001
	2005	146	4.8125	0.4960	0.0410	3.7	6.33	0.10	<0.0001
Y_5_	2003	105	4.8644	0.2823	0.0275	4.336	5.802	0.06	<0.0001
	2004	129	4.5932	1.1330	0.0175	3.19	6.61	0.25	<0.0001
	2005	146	4.5838	0.3326	0.0275	3.91	5.27	0.07	<0.0001
Z	2003	105	856.1210	241.0244	23.5216	371.69	1525.07	0.28	<0.0001
	2004	129	539.4421	220.5409	3.4152	139.65	1312.04	0.41	<0.0001
	2005	146	523.4232	225.8983	18.6955	130.15	1271.088	0.43	<0.0001

Y_1_, fertile tillers m^-2^; Y_2_, spikelets/fertile tillers; Y_3_, florets/spikelet; Y_4_, seed numbers/spikelet; Y_5_, seed weight (mg); Z, seed yield (kg hm^-2^).

**Table 2:** Pearson correlation coefficients of X_3_, X_2_, Y_1_–Y_5_, and Z.

	years	X_2_	Y_1_	Y_2_	Y_3_	Y_4_	Y_5_	Z
X_3_	2003	0.117**	0.136**	0.106**	0.129**	–0.036*	–0.062**	0.183**
	2004	0.117**	–0.052**	0.003	–0.020	–0.005	–0.047**	–0.022
	2005	0.117**	0.189**	0.039*	–0.007	–0.153**	–0.170**	0.274**
	2003–05	0.117**	–0.050**	–0.018	0.012	–0.004	–0.006	0.003
X_2_	2003	1	–0.052**	0.005	0.124**	–0.003	–0.095**	0.051**
	2004	1	0.101**	0.110**	–0.053**	–0.030	–0.076**	0.209**
	2005	1	–0.073**	–0.078**	0.018	–0.011	0.232**	0.075**
	2003–05	1	0.128**	0.081**	0.036**	–0.041**	–0.071**	0.170**
Y_1_	2003	–	1	0.103**	–0.124**	–0.066**	–0.255**	0.377**
	2004	–	1	0.119**	–0.106**	–0.082**	–0.084**	0.286**
	2005	–	1	0.052**	0.020	–0.338**	–0.589**	0.533**
	2003–05	–	1	0.085**	0.441**	–0.069**	–0.202**	0.125**
Y_2_	2003	–	–	1	–0.017	–0.016	–0.058**	0.129**
	2004	–	–	1	–0.028	–0.010	–0.029	0.119**
	2005	–	–	1	0.005	0.041*	–0.007	–0.001
	2003–05	–	–	1	–0.291**	–0.010	0.022*	0.172**
Y_3_	2003	–	–	–	1	0.019	0.106**	0.008
	2004	–	–	–	1	0.030	0.042*	0.010
	2005	–	–	–	1	0.022	0.010	0.002
	2003–05	–	–	–	1	0.011	–0.026*	–0.007
Y_4_	2003	–	–	–	–	1	0.026	–0.077**
	2004	–	–	–	–	1	–0.019	0.012
	2005	–	–	–	–	1	0.228**	–0.207**
	2003–05	–	–	–	–	1	0.001	–0.018
Y_5_	2003	–	–	–	–	–	1	–0.220**
	2004	–	–	–	–	–	1	–0.025
	2005	–	–	–	–	–	1	–0.299**
	2003–05	–	–	–	–	–	1	0.051**

F-values are shown for each year with statistical differences

*P<0.05

**P<0.01

***P<0.0001.

*N* = 105, 129, and 146 for years 2003, 2004, and 2005, respectively. X_3_, nitrogen fertilizer; X_2_, irrigation; Y_1_, fertile tillers/m^2^; Y_2_, spikelets/fertile tillers; Y_3_, florets/spikelet; Y_4_, seed number/spikelet; Y_5_, seed weight; Z, seed yield.

**Table 3:** Analysis of variance in models of Y_1_–Y_5_, Z, and irrigation (X_2_) with nitrogen (X_3_). Y_1_, fertile tillers/m^2^; Y_2_, spikelets/fertile tillers; Y_3_, florets/spikelet; Y_4_, seed numbers/spikelet; Y_5_, seed weight; and Z, seed yield.

Variable			Y_1_		Y_2_		Y_3_		Y_4_		Y_5_		Z	
	Df	Year	*F*	*Pr*	*F*	*Pr*	*F*	*Pr*	*F*	*Pr*	*F*	*Pr*	*F*	*Pr*
X_2_	6	2003	**8.036**	**<0.001**	1.521	0.167	18.245	<0.001	1.266	0.270	**42.956**	**<0.001**	**14.025**	**<0.001**
		2004	**8.627**	**<0.001**	1.860	0.084	0.362	0.903	1.325	0.242	**12.885**	**<0.001**	**15.154**	**<0.001**
		2005	**43.267**	**<0.001**	1.279	0.264	1.521	0.167	47.000	<0.001	**57.694**	**<0.001**	**15.397**	**<0.001**
		2003–05	**21.370**	**<0.001**	3.047	0.006	0.608	0.724	5.732	<0.001	**13.463**	**<0.001**	**9.943**	**<0.001**
X_3_	11	2003	**79.240**	**<0.001**	7.389	<0.001	41.009	<0.001	1.839	<0.05	**50.105**	**<0.001**	**133.819**	**<0.001**
		2004	**80.543**	**<0.001**	7.460	<0.001	6.845	<0.001	1.773	.053	**4.304**	**<0.001**	**46.550**	**<0.001**
		2005	**426.623**	**<0.001**	2.279	.009	7.389	<0.001	67.216	<0.001	**194.579**	**<0.001**	**51.567**	**<0.001**
		2003–05	**207.725**	**<0.001**	11.551	.000	2.879	<0.001	8.090	<0.001	**17.979**	**<0.001**	**108.375**	**<0.001**
X_2_×X_3_	11	2003	**5.836**	**<0.001**	2.339	<0.01	35.589	<0.001	1.848	<0.05	**32.156**	**<0.001**	**75.969**	**<0.001**
		2004	**5.821**	**<0.001**	2.634	<0.01	1.517	0.118	1.535	0.112	**21.204**	**<0.001**	**44.821**	**<0.001**
		2005	**22.704**	**<0.001**	1.497	0.125	2.339	<0.01	66.629	<0.001	**31.480**	**<0.001**	**45.627**	**<0.001**
		03–05	**15.384**	**<0.001**	3.202	0.000	1.399	0.166	7.798	<0.001	**24.458**	**<0.001**	**63.229**	**<0.001**

**Table 4:** Analysis of variance of the random effects from year (2003, 2004, or 2005), group (G, six experimental groups), and pair-wise comparisons of fertile tillers/m^2^ (Y_1_), spikelets/fertile tillers (Y_2_), florets/spikelet (Y_3_), seed numbers/spikelet (Y_4_), seed weight (Y_5_), and seed yield (Z). PROC MIXED ANOVA analysis was performed using SAS.

Variable	Y_1_		Y_2_		Y_3_		Y_4_		Y_5_		Z	
	*F*	*Pr*	*F*	*Pr*	*F*	*Pr*	*F*	*Pr*	*F*	*Pr*	*F*	*Pr*
G	3.65	0.057	0.29	0.411	1.07	0.143	–	–	1.37	0.086	0.78	0.217
X_2_×G	0.80	0.372	0.13	0.447	–	–	–	–	–	–	0.71	0.239
X_2_×Y	2.02	0.157	**2.53**	**<0.01**	**3.71**	**<0.001**	**3.46**	**<0.01**	**2.95**	**<0.01**	**2.69**	**<0.01**
Y	51.3	<0.001	1.20	0.116	1.22	0.111	1.22	0.1112	1.20	0.115	1.20	0.115
Y×G	**5.56**	**<0.01**	**2.46**	**<0.01**	**2.70**	**<0.01**	**2.63**	**<0.01**	**2.18**	**<0.05**	**2.48**	**<0.01**
X_3_×G	1.36	0.245	1.497	0.125	–	–	–	–	2.15	**0.016**	0.77	0.221
X_3_×Y	0.41	0.524	**3.05**	**<0.01**	**4.89**	**<0.001**	**4.46**	**<0.001**	**3.49**	**<0.01**	**1.399**	**<0.01**

### Irrigation and N fertilizer differentially affect seed yield

N fertilizer (X_3_) had a direct effect on all seed yield traits (Table 5). The total effect of X_3_ in order of absolute value from Y_1_–Y_5_ and Z was Z (0.835) > Y_1_ (0.590) > Y_5_ (–0.394) > Y_2_ (0.351) > Y_4_ (–0.260) > Y_3_ (0.091). The direct effect of X_3_ on Z was positive and strong (*P*<0.0001; highlighted in bold in Table 5), with coefficients of 0.180 in 2003, 0.214 in 2004, and 0.268 in 2005. Among the five seed yield components, N fertilizer affected seed yield (Z) most strongly. The direct effect of N (X_3_) on Y_1_ was positive and strong in all three experimental years (*P*<0.0001), with coefficients of 0.144 in 2003, 0.109 in 2004, and 0.201 in 2005.

**Table 5:** Path analyses showing indirect effects of N fertilizer (X_3_) and irrigation (X_2_) on fertile tillers/m^2^ (Y_1_), spikelets/fertile tillers (Y_2_), florets/spikelet (Y_3_), seed numbers/spikelet (Y_4_), seed weight (Y_5_), and seed yield (Z).

		Indirect effect via					
	Year	Y_1_	Y_2_	Y_3_	Y_4_	Y_5_	Z
X_2_→X_2_	2003	–0.069***	–0.070	0.110***	0.001	–0.089***	0.030
	2004	–0.065***	–0.010	–0.014	–0.001	–0.039*	–0.047**
	2005	–0.096***	–0.084***	0.019	0.007	0.255***	0.044*
	2003–05	–0.066***	–0.028**	0.008	0.001	0.003	**–**0.018
	**Total**	–0.296	–0.192	0.123	0.008	0.130	0.009
X_2_→X_3_	2003	0.017	0.013	0.014	**–**0.004	**–**0.006	0.021
	2004	0.013	0.013	**–**0.006	**–**0.004	**–**0.008	0.025
	2005	0.024	0.006	**–**0.002	**–**0.027	**–**0.035	0.047
	2003–05	0.024	0.010	0.004	**–**0.005	**–**0.008	0.020
	**Total**	0.078	0.042	0.010	**–**0.040	**–**0.057	0.113
X_3_→X_3_	2003	0.144***	0.107***	0.116***	**–**0.036*	–0.052**	0.180***
	2004	0.109***	0.111***	**–**0.051*	**–**0.030	–0.072***	0.214***
	2005	0.201***	0.049**	**–**0.009	**–**0.153***	–0.199***	0.268***
	2003–05	0.136***	0.084***	0.035***	**–**0.041**	–0.071***	0.173***
	**Total**	0.590	0.351	0.091	**–**0.260	**–**0.394	0.835
X_3_→X_2_	2003	–0.008	–0.008	0.013	0.000	–0.010	0.004
	2004	–0.008	–0.001	–0.002	0.000	–0.005	–0.005
	2005	–0.011	–0.010	0.003	0.001	0.045	0.005
	2003–05	–0.012	–0.003	0.001	0.000	0.001	–0.002
	**Total**	–0.039	–0.022	0.015	0.001	0.031	0.002

F-values are shown with statistical differences for each year

*P<0.05

**P<0.01

***P<0.0001.

Irrigation (X_2_) had a weak direct effect on the yield factors Y_2_, Y_3_, and Y_4_. By contrast, irrigation had a strong, highly significant, and direct effect on Y_1_, Y_5_, and Z in at least two experimental years (Table 5). Irrigation showed significantly negative correlation with Y_1_ in all three experimental years (*P*<0.0001), with coefficients of –0.069 in 2003, –0.065 in 2004, and –0.096 in 2005. Therefore, irrigation had the largest effect on Y_1_. Irrigation significantly affected Y_5_, with coefficients of –0.089 in 2003, –0.039 in 2004, and 0.255 in 2005. Irrigation had a strong, direct effect on Y_1_, Y_5_, and Z, and the total effect in order of absolute value was Y_1_ (–0.296) > Y_2_ (–0.192) > Y_5_ (0.130) > Y_3_ (0.123) > Z (0.009) > Y_4_ (0.008) (Table 5).

Path analysis was performed to determine indirect contributions between X_2_ and X_3_. The strongest positive indirect effect on Z was from the X_2_ via X_3_ path (the coefficient was 0.047 in 2005), and the second strongest indirect effect was on Y_5_ from the X_3_ through X_2_ path (0.045 in 2005). We observed an effect on Z by X_2_ via X_3_ (0.025 in 2004) and on Y_1_ by X_2_ via X_3_ (0.024 in 2005). The strongest negative indirect effects were on Z, Y_1_, and Y_5_ were by X_2_ via X_3_ (–0.035 in 2005), and the second strongest indirect effects were on Y_4_ by X_2_ via X_3_ (–0.027 in 2005). X_2_ via X_3_ effects on Y_3_ were positive in 2003 but negative and marginal in the other two experimental years. X_2_ via X_3_ effects on Y_1_, Y_2_, and Z were positive in all experimental years. X_2_ via X_3_ effects on Y_4_ and Y_5_ were negative in all three experimental years (Table 5). Comparison of the X_2_ via X_3_ and X_3_ via X_2_ paths indicated that N fertilizer had little effect on seed yield and seed yield factors through irrigation. For the X_2_ via X_3_ path, the order of the contributions with respect to absolute value and total direct effects were Z (0.113) > Y_1_ (0.078) > Y_5_ (–0.057) > Y_2_ (0.042) > Y_4_ (0.040) > Y_3_ (0.010), which was the same as the effect of N fertilizer (Table 5). By contrast, the effects of X_3_ via X_2_ were much smaller, and the absolute value order was Y_1_ (–0.039) > Y_5_ (0.031) >Y_2_ (–0.022) > Y_3_ (0.015) > Z (0.002) > Y_4_ (0.001), which was similar to the irrigation effect (Table 3).

### Antagonistic effects of irrigation and N fertilizer on Y_1_

**Fig 3:**
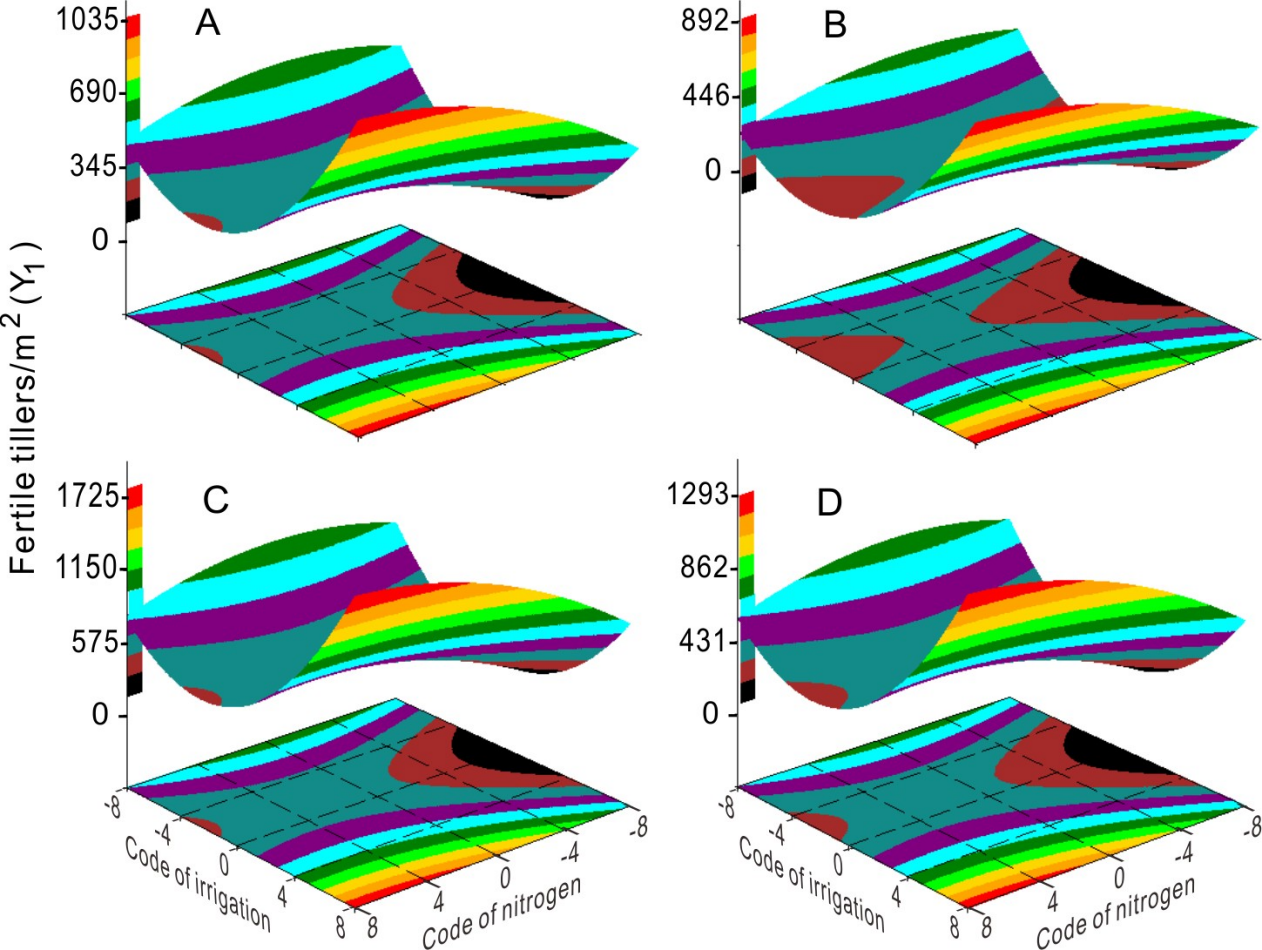
Response surface shows the coupling effects of irrigation and nitrogen fertilizer on fertile tillers/m^2^ (Y1) in 2003 (A), 2004 (B), 2005 (C), and 2003–2005 (D).

**Fig 4:**
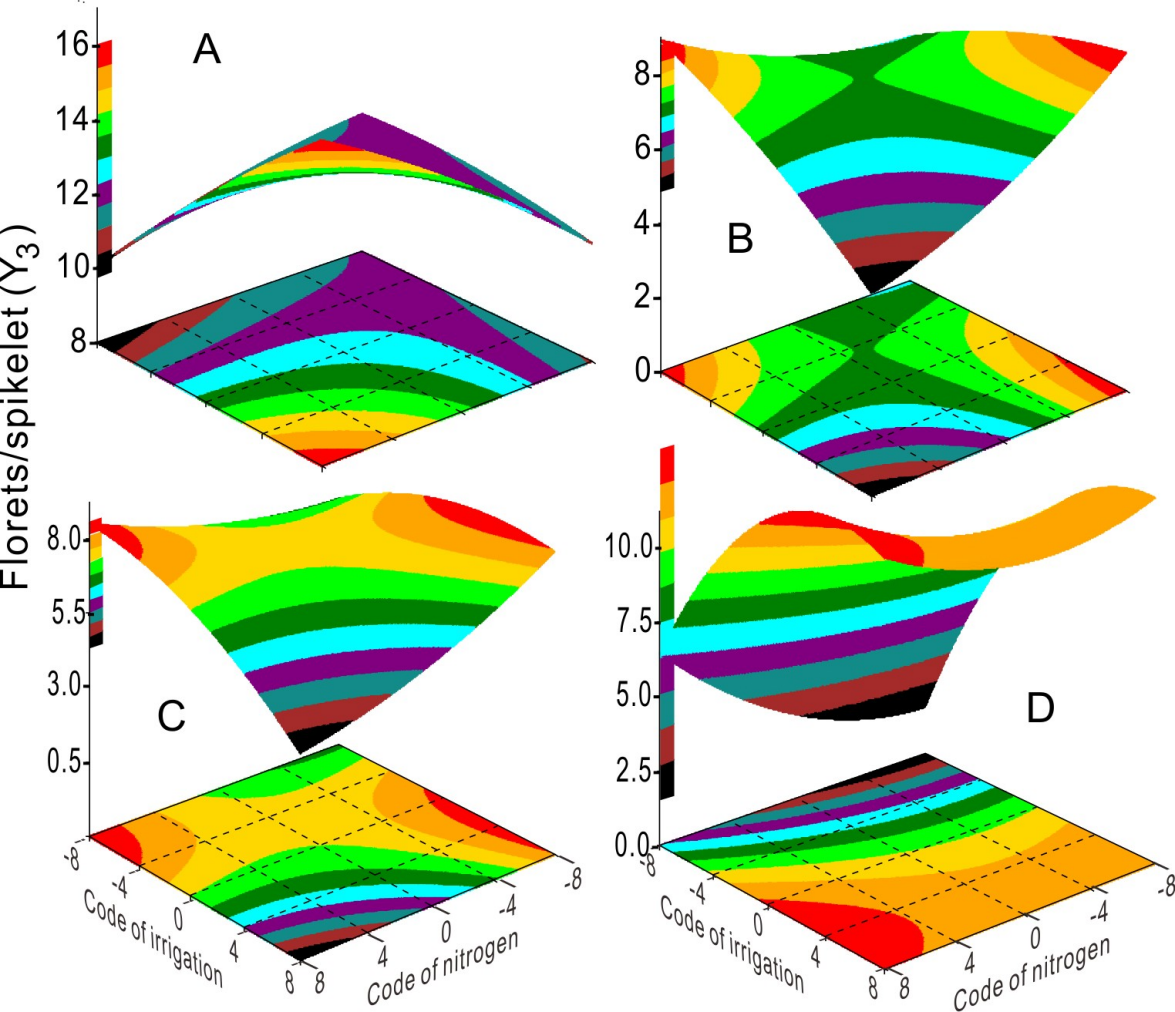
Response surface shows the coupling effects of irrigation and nitrogen fertilizer on florets/spikelet (Y3) for 2003 (A), 2004 (B), 2005 (C), and 2003–2005 (D).

Application of 0–360 kg ha^-1^ N fertilizer significantly reduced Y_1_ (fertile tiller) as irrigation increased. Under these conditions, irrigation determined Y_1_ (Fig 3) and lower irrigation was beneficial for the production of fertile tillers. The number of florets (Y_3_, Fig 4) were unaffected by high (Fig 4A and 4D) or low (Fig 4B and 4C) irrigation, and higher N application (360–480 kg ha^-1^) was beneficial for higher floret numbers (Y_3_, Fig 4). Similarly, seed yield was high under conditions of lower irrigation with higher N (Z, Fig 2A), or higher irrigation with higher N (Z, Fig 2B–2D).

**Fig 5:**
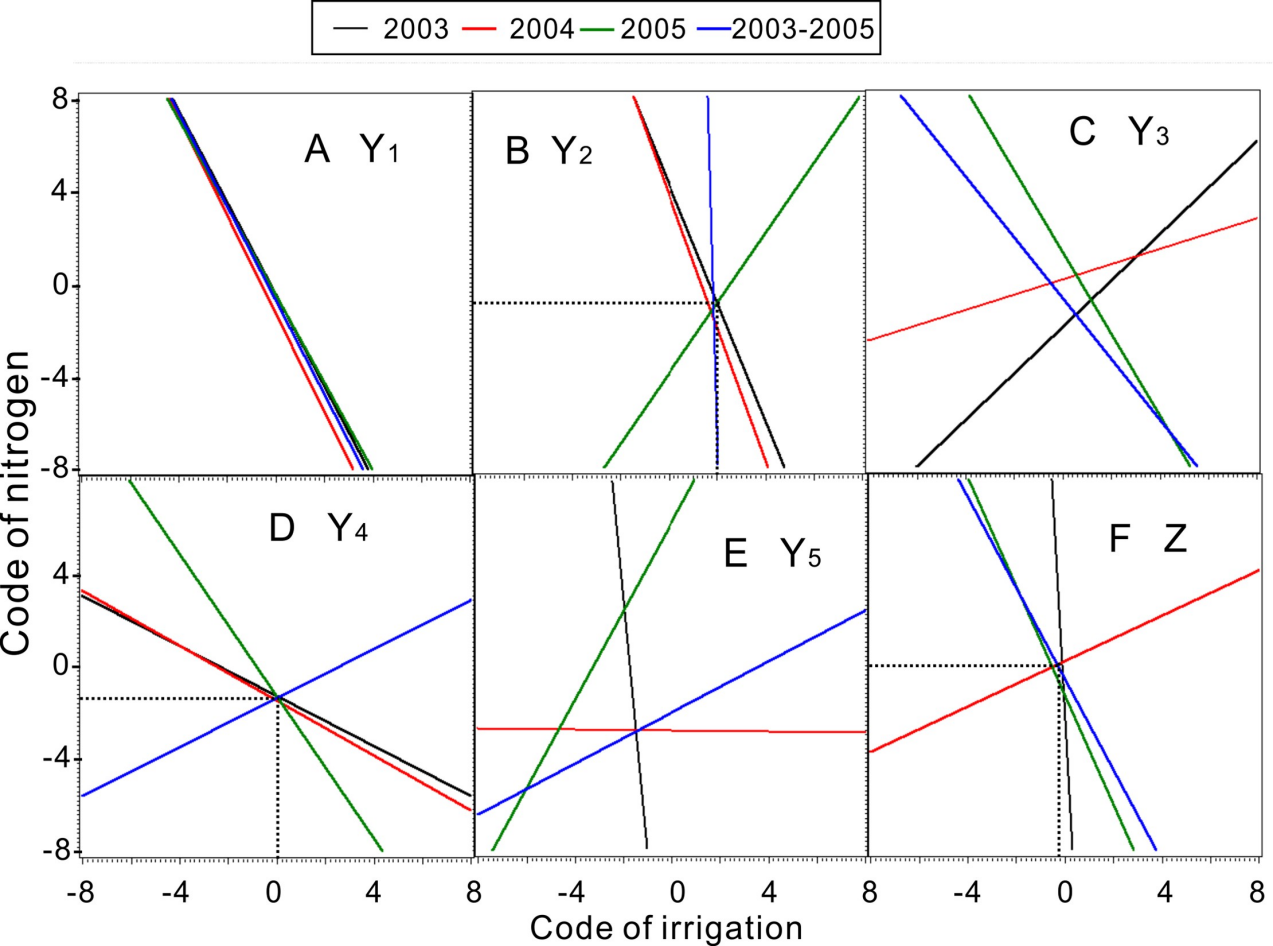
Ridgelines of the models for nitrogen fertilizer and irrigation effects on seed yield (F) and seed yield components (A through E).

The antagonistic and synergistic effects of irrigation and N application rate on Z and Y variables were investigated using pairwise quadratic regression models, shown in ridgelines (Fig 5). The range lines (equivalent effects) of Y_1_ for all three experimental years nearly overlapped (Fig 5A). The linear model for N effects on irrigation was obtained by averaging the four slopes from all three experimental years (–1.98 in 2003, –2.11 in 2004, –1.89 in 2005, and –2.02 in 2003–2005) and the constants.

$$X_{3}=-1.996\times X_{2}-0.615\;(R^{2}=0.999,F=6012.485,\;\mathrm{and}\;P<0.0001)$$ (4)

With codes converted into applications of N and irrigation,

$$X_{3}=635.2-4.23\times X_{2}\;(R^{2}=1.000,F=532.215,\;\mathrm{and}\;P<0.0001)$$ (5)

where X_3_ is N (kg hm^–2^) and X_2_ is irrigation (mm).

With summed average annual precipitation of 55 mm at the experimental location,

$$X_{3}=867.6-4.23\times X_{2}\;(R^{2}=1.000,F=446.318,\;\mathrm{and}\;P<0.0001)$$ (6)

X_2_ was assigned new means for the total annual water requirement (precipitation and irrigation). The linear model results suggested that irrigation and N application rate had antagonistic effects on Y_1_. X_2_ effects were more dynamic than those of X_3_ (Fig 5A), which was caused primarily by feedforward compensation at the biological level and by limited soil nutrients.

### Optimized N and irrigation rates for Y_2_, Y_4_, and Z

The ridgelines of Y_2_, Y_4_, and Z nearly crossed at one point for all years (Fig 5B, 5D and 5F). We calculated these coordinates to determine that optimal ranges of N application and irrigation were 120 kg/ha and 146 mm for spikelet/fertile tillers (Y_2_), 108 kg/ha and 119 mm for seed numbers/spikelet (Y_4_), and 144 kg/ha and 115 mm for seed yield (Z), respectively (Fig 5B, 5D and 5F). The response surface and contour charts for the coupled effects of N fertilizer and irrigation on seed yield displayed two different patterns: one in which seed yield increased with higher irrigation and lower N supply (Fig 2A), and another in which seed yield increased when N fertilizer application and irrigation both increased (Fig 2B–2D). Two lines in the same direction on the ridgeline graph indicated that N fertilization and irrigation for the years 2004 and 2003–2005 had antagonistic effects on Y_2_ (Fig 5B) at *k*<0 in the ridgelines. The green lines show different directions, indicating antagonistic effects on Y_2_ in the ridgelines at *k*>0 (Fig 5B). The red, black, and green lines in the same directions also indicated that irrigation and N supply had antagonistic effects on Y_4_ in each year (Fig 5D) at *k*<0 in the ridgelines. The blue, black, and green lines in the same direction as the green line of Y_4_ indicated antagonistic effects on seed yield Z at *k*<0 (Fig 5). Conversely, the lines for 2004 (red) displayed an orientation with the red lines of Y_4_ and Y_2_, which indicated synergistic effects on Z (Fig 5) at *k*>0.

All the seed weight (Y_5_) lines extended in different directions from those of florets/spikelet (Y_3_) in each year (Fig 5C and 5E). For example, the Y_5_ lines in 2005 (green) displayed synergetic effects between irrigation and N supply (*k*>0), but the Y_3_ lines in 2005 (green) displayed antagonistic effects (*k*<0) between irrigation and N supply. All other ridgelines in each year display the same phenomenon (Fig 5C and 5E). Thus, the response surfaces in 2003 and 2005 show the highest floret growth with higher N application combined with increased irrigation (Fig 4A and 4D). By contrast, the response surface plots in 2004 and 2003–2005 show better growth at higher irrigation levels with lower N application, or else some properties gradually decreased as the irrigation and N supply increased (Fig 4B and 4C). All Y_5_ ridgelines almost crossed at one point, except for those in 2005 (green) (Fig 5E). The coordinates at the intersection of irrigation and N application levels were –1.5 to –1.39 and –2.87 to –2.8, respectively. The effects of irrigation, N fertilizer levels, and their interactions on Y_1_, Y_2_, Y_3_, Y_4_, Y_5_, and Z as determined by ANOVA are presented in S2 Fig.

## Discussion

Seed yield is primarily influenced by climate conditions, environmental factors, and extreme weather events [43], especially during the early seed development stage [44]. We obtained precipitation, temperature, and sunlight data during the crop-growing period from March to early September for the years 2003–2005 from the Jiuquan Meteorological Observatory of Gansu Province, China. Temperature and precipitation data during the crop growing seasons were close to the average values for the past ten years in the experimental study location (S14 Table). Thus, the field conditions during our study accurately represented the average field conditions. Our experimental conditions tested irrigation and N fertilization because these are the major limiting factors in modern agriculture. Water deficits during the plant reproductive stage often reduce crop yields by leading to reduced seed numbers [45], and water stress during seed filling reduces seed size [46]. Sufficient water during the growing season can visibly increase crop yield in drought-prone areas [47]. Conversely, heat stress during flowering reduces crop yields, indicating that temperature is important for yield outcomes during the flowering stage [48].

We conducted a three-year study that tested 18 levels of N fertilizer and 11 levels of irrigation (S4 Table) using large sample sizes (S3 Table). The results revealed differences in the magnitudes of the yield components. This observation suggests that N and water differentially affect numerous growth parameters under different rainfall and temperature conditions, including organ development, fertilization, seed formation, and seed development. Table 1 presents the differences in the magnitudes of seed yield components during 2003–2005. Seed yield ranged from 130 to 1525 kg ha^-1^ during the three years (Table 1). The mean seed yield in 2003 was almost 1.5-fold higher than that in 2004 or 2005. This may be explained by the fact that irrigation during the seed growing stage in grasses mitigates the negative effect of high temperatures. Precipitation in the seed growing stage of 2004 was higher than that in other years, which caused poor pollination, lodging, and an increase in common diseases, and may have resulted in poor translocation during the seed-filling period. The temperature affected crop productivity in conjunction with N availability. In the seed growing period, The temperature was lower during the seed growing period in 2003 than in 2004 or 2005 (S1 Fig). Thus, water and fertilizer levels had to be adjusted according to the temperature conditions. High seasonal temperatures can reduce the crop yield potential and increase mineralization [49]. Higher N levels reduced the number of degenerated spikelets that occurred under high-temperature conditions, and spikelets per panicle were positively correlated with N treatment levels under high temperatures. These results confirmed that increasing N application levels could alleviate crop yield losses caused by high temperatures [50]. These combined results indicate that the N supply should be adjusted accordingly under heat stress conditions.

Both excessive N and insufficient N have substantial effects on crop yield. Excessive amounts of ammonium N is toxic because it can inhibit Ca^2+^, Mg^2+^, and K^+^ absorption in plants [51]. The high variations in seed yield and seed yield components observed during the experiment from 2003–2005 (Table 1) may have been caused by interactions between experimental treatments and climate conditions, stand age divergence, air temperature differences, or combinations thereof. Thus, our results provide evidence of the interacting effects of climate factors, N fertilization, and irrigation on crop production in grasses.

This study evaluated the synergic and antagonistic effects of irrigation and N fertilization on seed yield and associated components. The irrigation level was positively correlated with applied N rates (Table 2), which was likely due to the increased plant canopy and high water absorption rate. Irrigation had a significant effect on the production of fertile tillers, seed weight, and seed yield (Tables 2 and 3). A previous study reported that water deficit affected the progression to plant maturity more than other growth stages and reduced the 1,000-seed weight [52], which is likely due to reduced assimilate uptake, storage, transfer, and half-filling of seeds [25, 53]. Our results show that seed yield was more sensitive to N fertilization than irrigation (Tables 2 and 3). The effect of N on seed yield may be indirect, as it also affects leaf area expansion, photosynthesis [54, 55], dry matter translocation [56], and sink capacity [57]. N application can increase leaf area expansion and photosynthesis in bermudagrass (*Cynodon dactylon* L.) [22] and creeping bentgrass (*Agrostis stolonifera* L.) [23], and these effects may propagate to affect seed yield components. Our study is consistent with other studies that report interacting effects of water and N fertilizer on crop yield [9]. In our study, increasing the rate of N application under high irrigation levels (148 mm) greatly increased seed yield (Fig 2). By contrast, high nitrogen levels reduced seed yield, but reducing irrigation levels under high nitrogen levels increased seed yield.

Medium irrigation levels resulted in substantial reductions in seed yield in our study. These irrigation levels enhanced leaf area, photosynthetic rate, internode length, internode number, and water transpiration rates. Thus, water is used primarily for vegetative growth rather than reproductive growth. Insufficient irrigation creates an ideal environment for insects, reduces vegetative growth, increases pollination, and promotes reproductive growth of crops. These results are consistent with Liebig's law of the minimum, which states that growth is dictated by the scarcest resource (limiting factor). Therefore, increasing either N fertilization or irrigation will only affect the crop’s vegetative growth, not its reproductive growth. Some studies have reported that high N application rates under severe drought stress accelerate water stress and may cause plant death [14]. Similarly, we found that irrigation and N application rate had antagonistic effects on fertile tillers, spikelets, and seed numbers (Fig 5A, 5B and 5D).

Plant reproductive growth begins with floral induction, which depends primarily on the growth conditions [14]. We found that the responses of fertile tillers to interactions between N and irrigation had linear superposition in all three years of our study, both in individual years and when combining years (Fig 5A), although the experimental and climatic conditions varied. We synthesized these responses into a steady-state algorithmic model: X_3_ = 867.6−4.23×X_2_ (R^2^ = 0.988). The converse of this generic result suggests that irrigation and N application cause a switch from vegetative to reproductive growth. Specifically, this model suggests that fertile tiller growth and the switch to reproductive growth may be favored by irrigation at 0 mm or by extremely poor soil N conditions [58]. Because water and N absorbed from the soil are necessary for plant growth, these elements are indispensable for yield components. These combined results indicate that a smooth transition from vegetative growth to reproductive growth can be managed by avoiding excessive water and fertilizer application in mature crop plants.

N is important for maximum crop productivity, and achieving optimal levels of N and water is crucial to maximize crop production [59, 60]. In the presence of adequate water, increasing N application rates increased seed yield and yield components [61]. Another study reported significant interactions between N and water levels [62], similar to the results of our study. N fertilization can compensate for crop yield reductions caused by insufficient soil moisture. Sufficient soil moisture allows the amount of N fertilizer to be reduced; thus, high and stable crop yields can be maintained by managing the levels of water and fertilizer [59–61]. In our study, ridgeline analyses in all three years showed equivalent effects of irrigation and N application on spikelets (Fig 5B), seed numbers (Fig 5D), and seed yields (Fig 5F), and the lines almost crossed at one point. These results suggest that optimal levels of N and irrigation can be achieved for crops. Our observations suggest that the main cause of synergy between irrigation and N [63] (Fig 5E) may be due to high precipitation in March 2005 (S1 Fig). Seed weight is considered as genetically fixed and unresponsive to changes in N input [64]. Thus, an increase in seed number rather than seed weight may improve seed yield [44]. The highest seed yields and seed numbers observed in our study occurred in 2003 (Table 1). Considering the coupled effects of irrigation and N in our study, we conclude that seed weight was affected primarily by N in 2003 and by irrigation in 2004 (Fig 5E). Our observations of excellent crop responses to irrigation or N in the quadratic two-variable regression models (Table 6) were consistent with previous studies [59]. The critical values at the stationary points in these models (*P*<0.0001) indicated their optimal values. When water was not limiting, we obtained the highest seed yield at >327 kg N/ha and the highest fertile tiller production at 95.92 kg N/ha. Other studies reported optimum yields at 96 kg N/ha [65], 150 kg N/ha [64], and 224 kg N/ha [8].

**Table 6:** Coefficients of pairwise models. X_3_, nitrogen fertilizer; X_2_, irrigation; Y_1_, fertile tillers/m^2^; Y_2_, spikelets/fertile tillers; Y_3_, florets/spikelet; Y_4_, seed numbers/spikelet; Y_5_, seed weight; Z, seed yield.

		X_2_^2^	X_3_^2^	X_2_×X_3_	X_2_	X_3_	Constant	*F* Value	*P*>*F*
2003	Y_1_	7.140	–1.848	2.337	16.859	14.000	300.549	64.257	<0.0001
	Y_2_	0.047	–0.024	–0.008	0.105	0.273	19.319	12.727	<0.0001
	Y_3_	–0.004	–0.004	0.027	0.167	0.098	12.833	19.886	<0.0001
	Y_4_	–0.026	0.007	–0.029	–0.108	–0.053	4.585	5.611	<0.0001
	Y_5_	–0.022	0.002	0.000	–0.088	–0.011	4.850	36.326	<0.0001
	Z	10.163	–3.299	–5.233	30.980	27.559	888.178	67.180	<0.0001
2004	Y_1_	8.092	–1.89	2.64	20.55	12.27	294.44	64.01	<0.0001
	Y_2_	0.057	–0.026	–0.009	0.125	0.286	19.361	14.165	<0.0001
	Y_3_	–0.005	0.008	–0.023	–0.057	–0.061	7.290	5.758	<0.0001
	Y_4_	–0.027	0.008	–0.028	–0.114	–0.049	4.549	5.642	<0.0001
	Y_5_	0.047	0.004	0.093	0.203	–0.036	4.453	27.948	<0.0001
	Z	3.565	0.844	12.289	16.156	18.217	565.844	45.109	<0.0001
2005	Y_1_	11.899	–3.081	3.895	28.099	23.334	500.915	139.115	<0.0001
	Y_2_	0.024	0.005	–0.065	–0.245	0.047	15.228	6.315	<0.0001
	Y_3_	0.014	–0.002	–0.038	0.034	–0.013	7.44	1.12	0.349
	Y_4_	0.026	0.007	–0.029	–0.108	–0.052	4.586	120.453	<0.0001
	Y_5_	0.014	0.004	–0.014	0.095	–0.041	4.484	102.169	<0.0001
	Z	10.59	–2.08	3.4	44.92	34.97	1092.5	74.13	<0.0001
2003–05	Y_1_	7.69	–1.89	2.51	18.86	13.30	301	126.97	<0.0001
	Y_2_	0.043	–0.015	–0.027	–0.005	0.202	17.969	20.695	<0.0001
	Y_3_	–0.005	–0.002	–0.002	0.053	0.017	10.02	1.46	0.201
	Y_4_	–0.026	0.007	–0.029	–0.110	–0.051	4.573	24.739	<0.0001
	Y_5_	0.043	0.004	0.087	0.191	–0.036	4.466	27.820	<0.0001
	Z	6.92	–1.24	3.53	23.92	23.09	733	70.381	<0.0001

This work also investigated interaction pathways for irrigation and N fertilization. Plant growth and photosynthetic capacity increase with increasing N supply [66]. The primary physiological response of plants to various N fertilization levels involves osmotic adjustment of NH_4_^+^ accumulation in the vacuole and compatible solute synthesis in the cytosol [67]. Crops grown under high levels of water and fertilizer usually accumulate inorganic ions in their vacuoles to reduce the cell water potential, because the energy consumed to absorb inorganic ions is far less than that required to synthesize organic compounds [68]. Ultimately, intracellular water levels decline and N ions increase in growth tissues. Our model for irrigation and N application is based on the combined results of this study and corroborating data in other publications [69, 70].

## Conclusions

This study produced a linear model for optimal N and irrigation application rates in western wheatgrass seed crops under specific environmental conditions. Water is a limiting factor in arid regions, and the optimal N application rate is coupled to the amount of water. Adjusting N levels to satisfy grass requirements under the given precipitation/irrigation levels should produce a beneficial synergy that optimizes seed yield.

1. Our results indicated that N fertilizer had a greater effect on seed yield and yield components than irrigation. Fertile tillers m^-2^ (Y_1_) had the strongest positive effect on seed yield (Z), whereas seed weight (Y_5_) had a negative effect on Z. Z, Y_1_, and Y_5_ were the characters that were most affected by N fertilizer and irrigation, and were positively correlated with N supply and negatively correlated with irrigation.
2. The interactions between N and irrigation display antagonistic effects on fertile tillers, and were synthesized into a steady-state algorithmic model: X_3_ = 867.6−4.23×X_2_ (R^2^ = 0.988, F = Infty and *P*<0.0001).
3. Our combined results indicate that the optimal amounts of N fertilizer and irrigation are 156 kg ha^-1^ + 115 mm for seed yield, 120 kg ha^-1^ + 146 mm for spikelets/fertile tillers, and 108 kg ha^-1^ + 119 mm for seed numbers/spikelets, respectively. These optimal values reduce the negative environmental effects of excessive N application, improve crop yields, and conserve agricultural inputs for *Pascopyrum smithii* growth in rid regions.

## Supporting information

S1 Table Nutrient contents in experimental soil used to grow *Pascopyrum smithii*. (DOCX)

S2 Table Experimental field design and factors. (DOCX)

S3 Table Sample size for measuring Y_1_ to Y_5_ and Z in each experimental plot and six groups of *Pascopyrum smithii*. (DOCX)

S4 Table Coding values of X_1_ to X_5_ and their corresponding usage in the six experimental designs. (DOCX)

S5 Table Compound matrix of L_8_ (4×2^4^) orthogonal design. (DOCX)

S6 Table Two-dimensional (2D) optimum design (1) (nitrogen and phosphorus). (DOCX)

S7 Table 2D optimum design (2) (nitrogen and phosphorus). (DOCX)

S8 Table Unique-factor orthogonal design. (DOCX)

S9 Table Compounding matrix of unique-factor orthogonal design. (DOCX)

S10 Table Bin-factor orthogonal contract blocks. (DOCX)

S11 Table Compound matrix of bin-factor orthogonal contract design. (DOCX)

S12 Table Tri-factor orthogonal rotary design. (DOCX)

S13 Table Unique-factor orthogonal design [L_8_ (41×2^4^)]. (DOCX)

S14 Table Monthly precipitation (mm) and average temperature (°C) at the China Agricultural University Grassland Research Station located in the Hexi Corridor, Jiuquan, Gansu Province, from 2003 to 2005. (DOCX)

S1 Fig Monthly rainfall and average air temperature from March to August for 2003, 2004, and 2005 at the research station in Jiuquan, Gansu Province, China. The meteorological station in Jiuquan provided these data.
(DOCX)

S2 Fig Comparison of fertile tillers/m^2^, spikelet/fertile tillers, florets/spikelet, seed numbers/spikelet, seed weight (mg), and seed yield (kg/km^2^) of western wheatgrass plants grown in the field under different nitrogen (N) and water conditions. The 18 treatments of N fertilizer ranged from 0 to 480 kg/ha [low N (LN) was 0, 44, 66, 88, 90, and 100 kg/ha; middle N (MN) was 107, 110, 120, 132, 150, and 153 kg/ha; and high N (HN) was 176, 180, 201, 210, 335, and 480 kg/ha]. The 11 levels of irrigation (X_2_) ranged from 0 to 148.1 mm [low water (LW) was 0, 52.78, 78, and 90.2 mm; middle water (MW) was 91, 104.1, 104.7, and 119.2 mm; and high water (HW) was 130, 133.6, and 148.1 mm].
(DOCX)
