# Supplementary material for: Optimizing irrigation and nitrogen fertilization for seed yield in western wheatgrass [Pascopyrum smithii (Rydb.) Á. Löve] using a large multi-factorial field design
Source: PLoS One. 2019 Jun 26;14(6):e0218599. doi: 10.1371/journal.pone.0218599 (PMC6594676; doi:10.1371/journal.pone.0218599)
Supplement: S1 Table — (DOCX) [file pone.0218599.s001.docx]

**Supporting Information**

**Table S1 The basic nutrient of experimental soil of *Pascopyrum smithii* Schreb.**

| Depth | PH | O.C. | T.S. | NH_4_^+^ | NO_3_^-^ | A.N. | A.P. | A.K. | T.N. | T.P. | T.K. |
| --- | --- | --- | --- | --- | --- | --- | --- | --- | --- | --- | --- |
| cm |  | g kg^-1^ | g kg^-1^ | mg kg^-1^ | mg kg^-1^ | mg kg^-1^ | mg kg^-1^ | mg kg^-1^ | g kg^-1^ | g kg^-1^ | g kg^-1^ |
| 0-20 | 8.39 | 10.32 | 4.88 | 32.32 | 20.09 | 118.30 | 36.56 | 130.30 | 0.764 | 0.814 | 12.52 |
| 20-40 | 8.30 | 10.33 | 7.69 | 31.09 | 12.33 | 90.88 | 18.24 | 127.00 | 0.744 | 0.733 | 11.82 |
| 40-60 | 8.41 | 7.23 | 7.50 | 37.49 | 10.26 | 80.99 | 16.42 | 148.40 | 0.441 | 0.700 | 13.51 |

Depth: Soil Depth, O.C. : Organic matter, T.S.: Total soli, NH4^+^ : Ammonium nitrogen, NO_3_^-^: Nitrate nitrogen, A.N.: Alkali hydrolysable nitrogen, A.P.: Available phosphorus, A.K.: Available potassium, T.N.: Total nitrogen, T.P.: Total phosphorus, T.K.: Total potassium.
