# Supplementary material for: Optimizing irrigation and nitrogen fertilization for seed yield in western wheatgrass [Pascopyrum smithii (Rydb.) Á. Löve] using a large multi-factorial field design
Source: PLoS One. 2019 Jun 26;14(6):e0218599. doi: 10.1371/journal.pone.0218599 (PMC6594676; doi:10.1371/journal.pone.0218599)
Supplement: S2 Table — (DOCX) [file pone.0218599.s002.docx]

**Supporting Information**

**Table S2 | Field experimental design and factors in gramineous forage**.

| Field experimental design groups | Matrices applied | Experimental factors | Repeat | plots | Matrices | Name |
| --- | --- | --- | --- | --- | --- | --- |
| A. 2-D-optimum design (1)^a^ | 2-D-optimum matrix | 2 (X_3,_ X_4_) | 3 | 18 | **Table S6** | **D16** |
| B. 2-D-optimum design (2)^a^ | 2-D-optimum matrix | 2 (X_3,_ X_4_) | 1 | 6 | **Table S7** | **D6** |
| C. Unique-factor orthogonal design | Compound matrix | 5 (X_1_-X_5_) | 1 | 36 | **Table S8, S9** | **Ww36** |
| D. Bin-factor orthogonal contract plots | Compound matrix | 2 (X_2_, X_3_+X_4_) | 1 | 22 | **Table S10, S11** | **Ew22** |
| E. Tri-factor orthogonal rotary design | Compounding matrix | 3 (X_1_, X_3_, X_6_) | 1 | 23 | **Table S12, S14** | **Se23** |
| F. Unique-factor orthogonal design | L_8_ (4^1^×2^4^) | 5 (X_1_, X_7_-X_10_) | 4 | 32 | **Table S13, S5** | **A32** |
| Control |  | -- | -- | 6-9 |  |  |
| Total |  | 10 (X_1_-X_6_) | -- | 143 |  |  |

^a^Applied N and P_2_O_5_ differently between design (1) and (2); In order, X_1_-X_10_ stand for: time of fertilizing, quantity of irrigation, applied nitrogen, applied P_2_O_5_, planted density, amount of spray plant regulator Paclobutrazol (PP333), irrigation time, density manipulation, time of cut post-harvest stubble, and burning post-harvest stubble.
