# Supplementary material for: Optimizing irrigation and nitrogen fertilization for seed yield in western wheatgrass [Pascopyrum smithii (Rydb.) Á. Löve] using a large multi-factorial field design
Source: PLoS One. 2019 Jun 26;14(6):e0218599. doi: 10.1371/journal.pone.0218599 (PMC6594676; doi:10.1371/journal.pone.0218599)
Supplement: S3 Table — (DOCX) [file pone.0218599.s003.docx]

**Supporting Information**

**Table S3. The sample size of Y_1_ to Y_5_, Z for each field experimental plot from combination of the six groups experiments on *Pascopyrum smithii* Schreb .**

| year | Sample size of plots (N) | | Sample size of each field experimental plot (N) | | | | | |
| --- | --- | --- | --- | --- | --- | --- | --- | --- |
|  |  |  | Fertile tillers m^-2^  Y_1_ | Spiklets/fertile tillers  Y_2_ | Florets/spiklet  Y_3_ | Seed numbers/spiklet  Y_4_ | Seed weight^a^  Y_5_ (mg) | Seed yield  Z (kg hm^-2^) |
| 2003 | 105 | | 10 | 51 | 27 | 24 | 10 | 4 |
| Total sample size(n) | | | 1050 | 5355 | 2835 | 2520 | 1050 | 420 |
| 2004 | 129 | | 10 | 30 | 30 | 30 | 10 | 4 |
| Total sample size(n) | | | 1290 | 3870 | 3870 | 3870 | 1290 | 516 |
| 2005 | | 146 | 10 | 30 | 30 | 30 | 10 | 4 |
| Total sample size(n) | | | 1460 | 4380 | 4380 | 4380 | 1460 | 584 |
| Three years totally(n) **380** | | | 3800 | 13605 | 11085 | 10770 | 3800 | 1520 |

^a^100-seed was take as one sample at a seed water content is at 7~10%, then 10 of the 100-seed sample in each plot were averaged to obtain one sample of seed weight (Y_5_) of the plot; the total sample size (n) of Y_5_ =10×105=1050 in 2003.

Total sample size (n) = Sample size of plots (N)×Sample size of each plot (n), e.g., the number of spikelets fertile tiller ^-1^ from 36 fertile tillers in each plot in 2003 was counted, then averaged as spikelets fertile tillers ^-1^ (Y_2_) of the plot, so, the total sample size (n) of Y_2_ =105×51=5355.
