# Supplementary material for: Optimizing irrigation and nitrogen fertilization for seed yield in western wheatgrass [Pascopyrum smithii (Rydb.) Á. Löve] using a large multi-factorial field design
Source: PLoS One. 2019 Jun 26;14(6):e0218599. doi: 10.1371/journal.pone.0218599 (PMC6594676; doi:10.1371/journal.pone.0218599)
Supplement: S4 Table — (DOCX) [file pone.0218599.s004.docx]

**Supporting Information**

**Table S4. Coding value of X_1_ to X_5_ and its corresponding usage combining the six experimental designs.**

| Factor | Time-of-fertilized(X_1_) | | Irrigation(X_2_) | | Nitrogcn (X_3_) | | Phosphorus (X_4_) | | Density-manipulation(X_5_) | |
| --- | --- | --- | --- | --- | --- | --- | --- | --- | --- | --- |
| **Treat**  **Level** | Level Code | Time | Level code | irrigation volume | Level code | Applied N(kg hm^-2^) | Level code | Applied P(kg hm^-2^) | Level code | Treatment |
| **1** | 0 | Autumn | -8.4 | 0 | -5 | 0 | -4.77 | 0 | 0 | Basic number |
| **2** | 1 | Tillering stage | -4.67 | 52.78 | -3.52 | 44 | -3.37 | 31 | -1 | 1/3 |
| **3** | 2 | Booting stage | -2.86 | 78 | -2.8 | 66 | -3 | 39 | -2 | 1/2 |
| **4** | 3 | Flowering stage | -2 | 90.2 | -2.06 | 88 | -2 | 61 | -2.5 | blank |
| **5** | 4 | Filling stage | -1.94 | 91 | -2 | 90 | -1.97 | 62 |  |  |
| **6** |  |  | -1.05 | 104.1 | -1.628 | 100 | -1.91 | 63 |  |  |
| **7** |  |  | -1 | 104.7 | -1.45 | 107 | -1.26 | 77 |  |  |
| **8** |  |  | 0 | 119.2 | -1.32 | 110 | -1 | 83 |  |  |
| **9** |  |  | 0.75 | 130 | -1 | 120 | -0.68 | 90 |  |  |
| **10** |  |  | 1 | 133.6 | -0.59 | 132 | -0.56 | 93 |  |  |
| **11** |  |  | 2 | 148.1 | 0 | 150 | 0 | 105 |  |  |
| **12** |  |  |  |  | 0.1 | 153 | 0.84 | 124 |  |  |
| **13** |  |  |  |  | 0.88 | 176 | 1 | 127 |  |  |
| **14** |  |  |  |  | 1 | 180 | 2 | 149 |  |  |
| **15** |  |  |  |  | 1.628 | 201 | 2.84 | 167 |  |  |
| **16** |  |  |  |  | 2 | 210 | 6.14 | 240 |  |  |
| **17** |  |  |  |  | 6.15 | 335 |  |  |  |  |
| **18** |  |  |  |  | 11 | 480 |  |  |  |  |

*1/2 means remove the 1/2 line at tillering stage; 1/3 means remove the 1/3 line at tillering stage.
