# Supplementary material for: Optimizing irrigation and nitrogen fertilization for seed yield in western wheatgrass [Pascopyrum smithii (Rydb.) Á. Löve] using a large multi-factorial field design
Source: PLoS One. 2019 Jun 26;14(6):e0218599. doi: 10.1371/journal.pone.0218599 (PMC6594676; doi:10.1371/journal.pone.0218599)
Supplement: S5 Table — (DOCX) [file pone.0218599.s005.docx]

**Supporting Information**

**Table S5. Compound Matrix of L_8_（4×2^4^）Orthogonal Design**

| Treatments  No. of block | Array 1  A | Array 2  B | Array 3  C | Array 4  D | Array 5  E |
| --- | --- | --- | --- | --- | --- |
| 1 | 1 | 1 | 1 | 1 | 1 |
| 2 | 1 | 2 | 2 | 2 | 2 |
| 3 | 2 | 1 | 1 | 2 | 2 |
| 4 | 2 | 2 | 2 | 1 | 1 |
| 5 | 3 | 1 | 2 | 1 | 2 |
| 6 | 3 | 2 | 1 | 2 | 1 |
| 7 | 4 | 1 | 2 | 2 | 1 |
| 8 | 4 | 2 | 1 | 1 | 2 |

Note: 4 repeat, 32 blocks total.
