# Supplementary material for: Optimizing irrigation and nitrogen fertilization for seed yield in western wheatgrass [Pascopyrum smithii (Rydb.) Á. Löve] using a large multi-factorial field design
Source: PLoS One. 2019 Jun 26;14(6):e0218599. doi: 10.1371/journal.pone.0218599 (PMC6594676; doi:10.1371/journal.pone.0218599)
Supplement: S6 Table — (DOCX) [file pone.0218599.s006.docx]

**Supporting Information**

**Table S6. A. 2-D optimum design (1) (Nitrogen and Phosphorus)**

| Treatment  No. of Blocks | Factor X_3_ (Nitrogen, N) | | Factor X_4_ (Phosphorus, P_2_O_5_) | |
| --- | --- | --- | --- | --- |
|  | Level Code Applied N (kg/ha) | | Level Code Applied P_2_O_5_ (kg/ha) | |
| 1, (7,13) | -1 | 0 | -1 | 0 |
| 2, (8,14) | 1 | 153 | -1 | 0 |
| 3, (9,15) | -1 | 0 | 1 | 90 |
| 4, (10,16) | -0.1315 | 66 | -0.1315 | 39 |
| 5, (11,17) | 1 | 153 | 0.3945 | 63 |
| 6, (12,18) | 0.3945 | 106.5 | 1 | 90 |

Three repeat, 18 blocks total, each with a 28 m^2^ area.
