# Supplementary material for: Optimizing irrigation and nitrogen fertilization for seed yield in western wheatgrass [Pascopyrum smithii (Rydb.) Á. Löve] using a large multi-factorial field design
Source: PLoS One. 2019 Jun 26;14(6):e0218599. doi: 10.1371/journal.pone.0218599 (PMC6594676; doi:10.1371/journal.pone.0218599)
Supplement: S7 Table — (DOCX) [file pone.0218599.s007.docx]

**Supporting Information**

**Table S7. B. 2-D-optimum design (2) (Nitrogen and Phosphorus)**

| Treatment  No. of Blocks | Factor X_3_ (Nitrogen, N) | | Factor X_4_ (Phosphorus, P_2_O_5_) | |
| --- | --- | --- | --- | --- |
|  | Level Code Applied N(kg ha^-1^) | | Level Code Applied P_2_O_5_(kg ha^-1^) | |
| 1 | -1 | 0 | -1 | 0 |
| 2 | 1 | 480 | -1 | 0 |
| 3 | -1 | 0 | 1 | 240 |
| 4 | -0.1315 | 208.9 | -0.1315 | 104 |
| 5 | 1 | 480 | 0.3945 | 167 |
| 6 | 0.3945 | 334.5 | 1 | 240 |

Six blocks total, each with a 28 m^2^ area.
