# Supplementary material for: Optimizing irrigation and nitrogen fertilization for seed yield in western wheatgrass [Pascopyrum smithii (Rydb.) Á. Löve] using a large multi-factorial field design
Source: PLoS One. 2019 Jun 26;14(6):e0218599. doi: 10.1371/journal.pone.0218599 (PMC6594676; doi:10.1371/journal.pone.0218599)
Supplement: S8 Table — (DOCX) [file pone.0218599.s008.docx]

**Supporting Information**

**Table S8. C. Unique-factor orthogonal design**

| Experimental Factor | Delta (∆) | (r = 2) Level-code-of-orthogonal design (r = 2)  –2 –1 0 1 2 | | | | |
| --- | --- | --- | --- | --- | --- | --- |
| Time of fertilization*(X_1_) | 1 | 1 | 2 | 3 | 4 | 5 |
| Irrigation-of-each-time (X_2_) | 14.4 (mm) | 90.4 | 104.8 | 119.2 | 133.6 | 148 |
| Applied N (X_3_) | 30 (kg ha^-1^) | 90 | 120 | 150 | 180 | 210 |
| Applied-P_2_O_5_ (X_4_) | 22 (kg ha^-1^) | 61 | 83 | 105 | 127 | 149 |
| Density manipulation (X_5_) | shoots ha^-1^ | 1/2 BD | 2/3 BD | BD | BD | BD |

*, the level codes for time of fertilization: 1, previous autumn; 2, tillering time; 3, time of stem elongation; 4, time of anthesis; 5, time of grain filling. Total of 36 blocks, each with a 28 m^2^ area.

BD, Basic Density.

and 153 kg/ha; and high N (HN): 176, 180, 201, 210, 335, and 480 kg/ha]. The 11 levels of irrigation (X_2_) ranged from 0 - 148.1 mm [low water (LW): 0, 52.78, 78, and 90.2 mm; middle water (MW): 91, 104.1, 104.7, and 119.2 mm; and high water (HW): 130, 133.6, and 148.1 mm].
