# Supplementary material for: Optimizing irrigation and nitrogen fertilization for seed yield in western wheatgrass [Pascopyrum smithii (Rydb.) Á. Löve] using a large multi-factorial field design
Source: PLoS One. 2019 Jun 26;14(6):e0218599. doi: 10.1371/journal.pone.0218599 (PMC6594676; doi:10.1371/journal.pone.0218599)
Supplement: S9 Table — (DOCX) [file pone.0218599.s009.docx]

**Supporting Information**

**Table S9. Compounding matrix of unique-factor orthogonal design**

| Treatments | X_0_ | X_1_ | X_2_ | X_3_ | X_4_ | X_5_ |
| --- | --- | --- | --- | --- | --- | --- |
| 1 | 1 | -1 | -1 | -1 | -1 | 1 |
| 2 | 1 | 1 | -1 | -1 | -1 | -1 |
| 3 | 1 | -1 | 1 | -1 | -1 | 1 |
| 4 | 1 | 1 | 1 | -1 | -1 | -1 |
| 5 | 1 | -1 | -1 | 1 | 1 | 1 |
| 6 | 1 | 1 | -1 | 1 | 1 | -1 |
| 7 | 1 | -1 | 1 | 1 | 1 | 1 |
| 8 | 1 | 1 | 1 | 1 | 1 | -1 |
| 9 | 1 | -1 | -1 | 1 | -1 | 1 |
| 10 | 1 | 1 | -1 | 1 | -1 | -1 |
| 11 | 1 | -1 | 1 | 1 | -1 | 1 |
| 12 | 1 | 1 | 1 | 1 | -1 | -1 |
| 13 | 1 | -1 | -1 | -1 | 1 | 1 |
| 14 | 1 | 1 | -1 | -1 | 1 | -1 |
| 15 | 1 | -1 | 1 | -1 | 1 | 1 |
| 16 | 1 | 1 | 1 | -1 | 1 | -1 |
| 17 | 1 | 0 | 0 | 0 | 0 | 0 |
| 18 | 1 | 0 | 0 | 0 | 0 | 0 |
| 19 | 1 | 0 | 0 | 0 | 0 | 0 |
| 20 | 1 | 0 | 0 | 0 | 0 | 0 |
| 21 | 1 | 0 | 0 | 0 | 0 | 0 |
| 22 | 1 | 0 | 0 | 0 | 0 | 0 |
| 23 | 1 | 0 | 0 | 0 | 0 | 0 |
| 24 | 1 | 0 | 0 | 0 | 0 | 0 |
| 25 | 1 | 0 | 0 | 0 | 0 | 0 |
| 26 | 1 | 0 | 0 | 0 | 0 | 0 |
| 27 | 1 | 2 | 0 | 0 | 0 | 0 |
| 28 | 1 | -2 | 0 | 0 | 0 | 0 |
| 29 | 1 | 0 | 2 | 0 | 0 | 0 |
| 30 | 1 | 0 | -2 | 0 | 0 | 0 |
| 31 | 1 | 0 | 0 | 2 | 0 | 0 |
| 32 | 1 | 0 | 0 | -2 | 0 | 0 |
| 33 | 1 | 0 | 0 | 0 | 2 | 0 |
| 34 | 1 | 0 | 0 | 0 | -2 | 0 |
| 35 | 1 | 0 | 0 | 0 | 0 | 2 |
| 36 | 1 | 0 | 0 | 0 | 0 | -2 |
