# Supplementary material for: Optimizing irrigation and nitrogen fertilization for seed yield in western wheatgrass [Pascopyrum smithii (Rydb.) Á. Löve] using a large multi-factorial field design
Source: PLoS One. 2019 Jun 26;14(6):e0218599. doi: 10.1371/journal.pone.0218599 (PMC6594676; doi:10.1371/journal.pone.0218599)
Supplement: S10 Table — (DOCX) [file pone.0218599.s010.docx]

**Supporting Information**

**Table S10. D. Bin-factor orthogonal contract blocks**

| Experimental Factor | (∆) | Level-code-of-orthogonal design (r = 2)  –3 –1 0 1 3 | | | | |
| --- | --- | --- | --- | --- | --- | --- |
| Irrigation-of each-time (X_1_) | 15.5 (mm) | 81.7 | 112.7 | 128.2 | 143.7 | 174.7 |
| (x_2_)  Applied N+P_2_O_5_* | 37.5  (kg ha^-1^) | 75  44.12+30.88 | 150  88.24+61.76 | 187.5  110.29+77.21 | 225  132.35+92.65 | 300  176.47+123.53 |

*Total of 22 blocks，each with a 28 m^2^ area. Applied fertilizer mixed according to N：P_2_O_5_ = 1：0.7
