# Supplementary material for: Optimizing irrigation and nitrogen fertilization for seed yield in western wheatgrass [Pascopyrum smithii (Rydb.) Á. Löve] using a large multi-factorial field design
Source: PLoS One. 2019 Jun 26;14(6):e0218599. doi: 10.1371/journal.pone.0218599 (PMC6594676; doi:10.1371/journal.pone.0218599)
Supplement: S11 Table — (DOCX) [file pone.0218599.s011.docx]

**Supporting Information**

**Table S11. Compound matrix of Bin-factor orthogonal contract design**

| Treatment | Irrigation (X_1_) | Applied N+ P_2_O_5_ (X_2_) |
| --- | --- | --- |
| 1 | 3 | 3 |
| 2 | 3 | 1 |
| 3 | 3 | -1 |
| 4 | 3 | -3 |
| 5 | 1 | 3 |
| 6 | 1 | 1 |
| 7 | 1 | -1 |
| 8 | 1 | -3 |
| 9 | -1 | 3 |
| 10 | -1 | 1 |
| 11 | -1 | -1 |
| 12 | -1 | -3 |
| 13 | -3 | 3 |
| 14 | -3 | 1 |
| 15 | -3 | -1 |
| 16 | -3 | -3 |
| 17 | 0 | 0 |
| 18 | 0 | 0 |
| 19 | 0 | 0 |
| 20 | 0 | 0 |
| 21 | 0 | 0 |
| 22 | 0 | 0 |
