# Supplementary material for: Optimizing irrigation and nitrogen fertilization for seed yield in western wheatgrass [Pascopyrum smithii (Rydb.) Á. Löve] using a large multi-factorial field design
Source: PLoS One. 2019 Jun 26;14(6):e0218599. doi: 10.1371/journal.pone.0218599 (PMC6594676; doi:10.1371/journal.pone.0218599)
Supplement: S12 Table — (DOCX) [file pone.0218599.s012.docx]

**Supporting Information**

**Table S12. E. Tri-factor orthogonal rotary design**

| Experimental Factor | Delta (∆) | (r = 2) Level-code-of-orthogonal-design (r = 2)  –1.682 –1 0 1 1.682 | | | | |
| --- | --- | --- | --- | --- | --- | --- |
| Density manipulation (X_1_) | shoots hm^-2^ | 1/2 BD | 2/3 BD | BD | BD | BD |
| Applied N (X_2_) | 30 (kg hm^-2^) | 99.5 | 120 | 150 | 180 | 200.5 |
| Plant regulator (PP333)  Paclobutrazol (PP333)*(X_3_) | a.i.0.08  kg ha^-1^ | 0.085 | 0.14 | 0.22 | 0.30 | 0.355 |

*Total of 23 blocks, each with a 28 m^2^ area. Paclobutrazol (PP333) was sprayed during stem elongation, twice with a week break.

BD, Basic Density.
