# Supplementary material for: Optimizing irrigation and nitrogen fertilization for seed yield in western wheatgrass [Pascopyrum smithii (Rydb.) Á. Löve] using a large multi-factorial field design
Source: PLoS One. 2019 Jun 26;14(6):e0218599. doi: 10.1371/journal.pone.0218599 (PMC6594676; doi:10.1371/journal.pone.0218599)
Supplement: S13 Table — (DOCX) [file pone.0218599.s013.docx]

**Supporting Information**

**Table S13. F. Unique-factor orthogonal design [L_8_（4^1^×2^4^）]**

| Factor  Level | A  Irrigation time | B  Planting Density | C ^1^  Time of  fertilization | D ^2^  Time of  Cut | E  Burning  Stubble |
| --- | --- | --- | --- | --- | --- |
| 1 | September + later Winter | 1/2 | Autumn | July | Burning |
| 2 | Post-harvest | 1 | Spring of the following year | September | Not burning |
| 3 | September |  |  |  |  |
| 4 | Later Winter |  |  |  |  |

**Note：**1, Applied nitrogen 150 kg ha^-1^，P_2_O_5_ 105 kg ha^-1^；2, Cutting with 25 mm stubble。
