# Supplementary material for: Optimizing irrigation and nitrogen fertilization for seed yield in western wheatgrass [Pascopyrum smithii (Rydb.) Á. Löve] using a large multi-factorial field design
Source: PLoS One. 2019 Jun 26;14(6):e0218599. doi: 10.1371/journal.pone.0218599 (PMC6594676; doi:10.1371/journal.pone.0218599)
Supplement: S14 Table — (DOCX) [file pone.0218599.s014.docx]

**Supporting Information**

**Table S14 Monthly precipitation (mm) and average temperature (°C) of the China Agricultural University Grassland Research Station located in the Hexi Corridor, Jiuquan, Gansu Province from 2003 to 2005**.

|  | Precipitation (mm) | | | Average temperature (℃) | | |
| --- | --- | --- | --- | --- | --- | --- |
| Month | 2003 | 2004 | 2005 | 2003 | 2004 | 2005 |
| Jan | 1.5 | 1.9 | 2.1 | -5.5 | -8.9 | -11.2 |
| Feb | 2.1 | 0.5 | 0 | -3.6 | -2.6 | -3.7 |
| Mar | 0.4 | 4.1 | 28.1 | 4.8 | 4.9 | 2.9 |
| Apr | 5 | 0.8 | 1.2 | 8.8 | 11.6 | 8.7 |
| May | 7.5 | 6.8 | 8.3 | 17 | 16.8 | 16.3 |
| Jun | 18.5 | 18.5 | 3.9 | 21 | 20.1 | 20.9 |
| Jul | 6.8 | 10.4 | 3.2 | 21.4 | 22.4 | 23.6 |
| Aug | 20 | 13.6 | 9.8 | 20.3 | 20.6 | 21.8 |
| Sep | 4.4 | 5.6 |  | 15.9 | 18.9 |  |
| Oct | 1.2 | 1.9 |  | 10.2 | 6.9 |  |
| Nov | 2.4 | 2.6 |  | 4.3 | 2.8 |  |
| Dec | 1.5 | 0 |  | -2.1 | 0.1 |  |
