# Supplementary material for: Optimizing irrigation and nitrogen fertilization for seed yield in western wheatgrass [Pascopyrum smithii (Rydb.) Á. Löve] using a large multi-factorial field design
Source: PLoS One. 2019 Jun 26;14(6):e0218599. doi: 10.1371/journal.pone.0218599 (PMC6594676; doi:10.1371/journal.pone.0218599)
Supplement: S1 Fig — The meteorological station in Jiuquan provided these data. (DOCX) [file pone.0218599.s015.docx]

**Supporting Information**


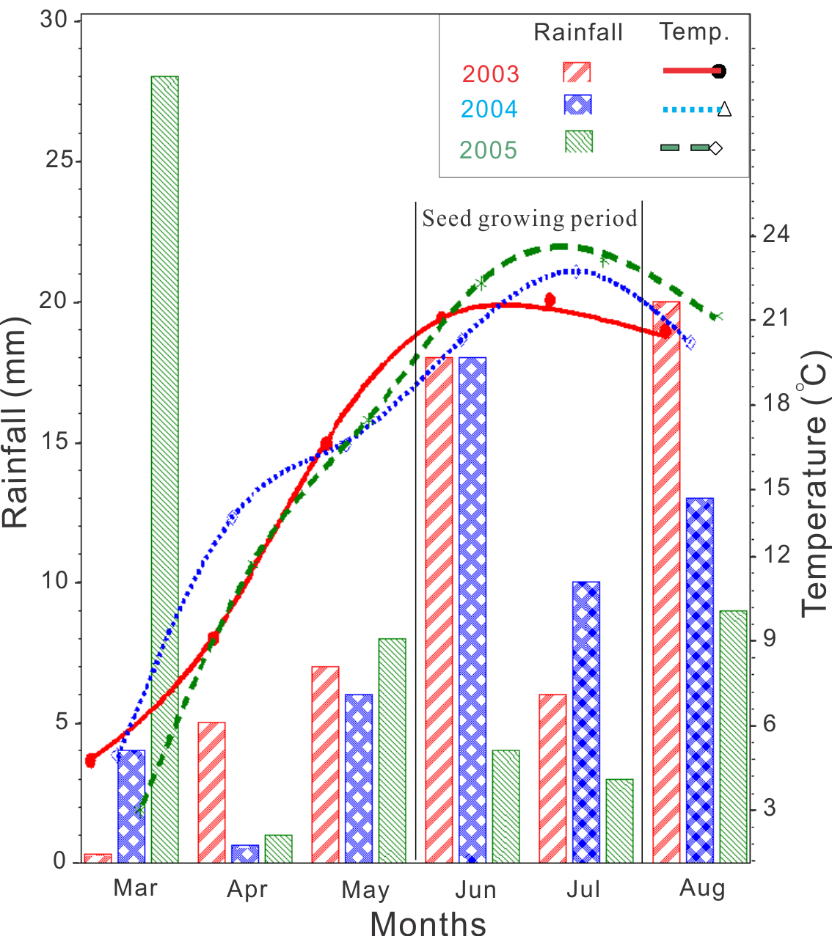


**S1 FIGURE. Monthly rainfall and average air temperature from March to August for 2003, 2004, and 2005 at the research location, in Jiuquan, Gansu province, China. The meteorological working station in Jiuquan, Gansu province, China, provided this data.**
