# Supplementary material for: Optimizing irrigation and nitrogen fertilization for seed yield in western wheatgrass [Pascopyrum smithii (Rydb.) Á. Löve] using a large multi-factorial field design
Source: PLoS One. 2019 Jun 26;14(6):e0218599. doi: 10.1371/journal.pone.0218599 (PMC6594676; doi:10.1371/journal.pone.0218599)
Supplement: S2 Fig — The 18 treatments of N fertilizer ranged from 0 to 480 kg/ha [low N (LN) was 0, 44, 66, 88, 90, and 100 kg/ha; middle N (MN) was 107, 110, 120, 132, 150, and 153 kg/ha; and high N (HN) was 176, 180, 201, 210, 335, and 480 kg/ha]. The 11 levels of irrigation (X2) ranged from 0 to 148.1 mm [low water (LW) was 0, 52.78, 78, and 90.2 mm; middle water (MW) was 91, 104.1, 104.7, and 119.2 mm; and high water (HW) was 130, 133.6, and 148.1 mm]. (DOCX) [file pone.0218599.s016.docx]

**Supplementary Information**


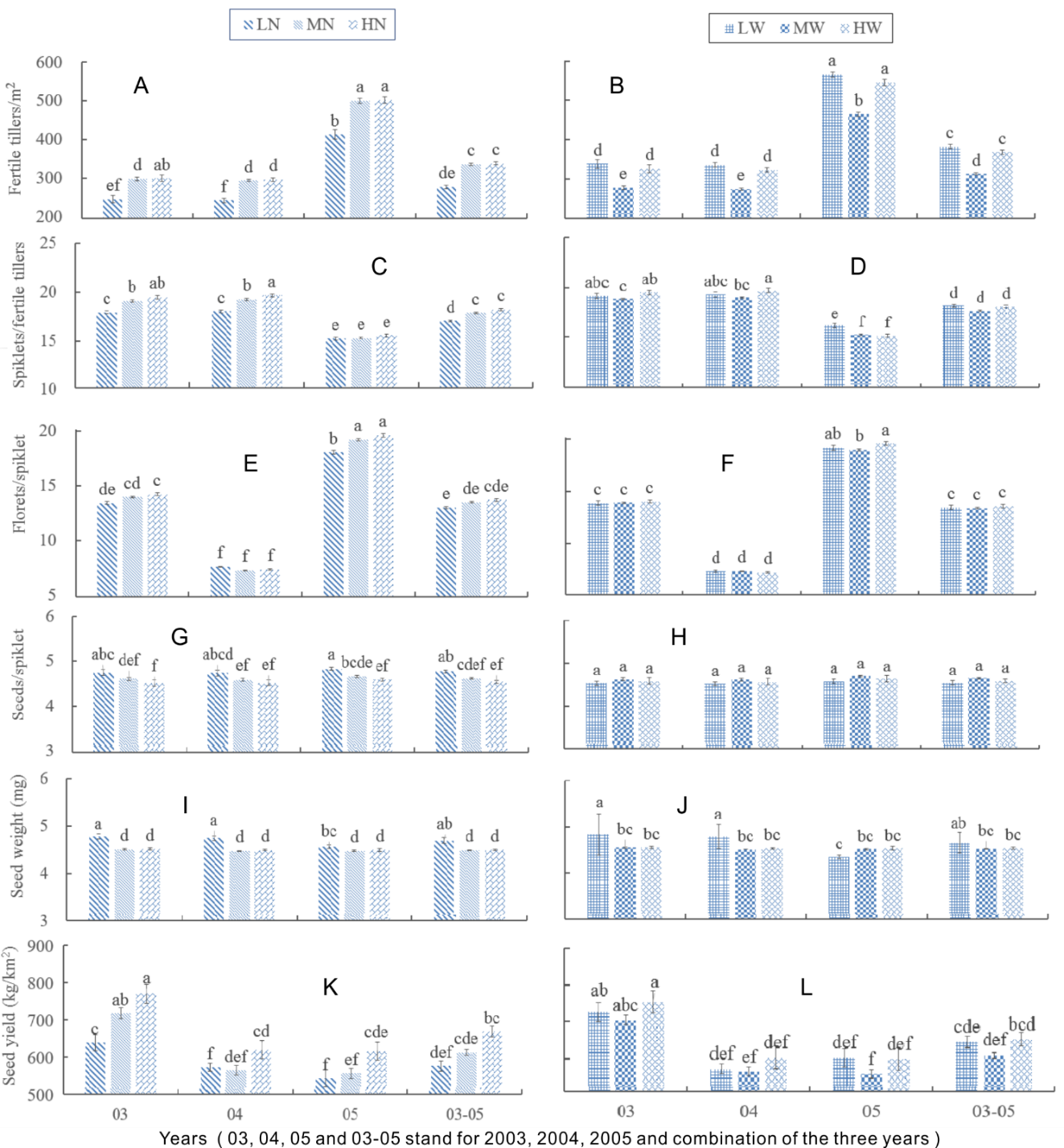


**S2 FIGURE. Comparison of fertile tillers/m^2^, spikelet/fertile tillers, florets/spikelet, seed numbers/spikelet, seed weight (mg), and seed yield (kg/km^2^) grown in the field under different nitrogen (N) and water conditions.** The 18 treatments of N fertilizer ranged from 0 to 480 kg/ha [low N (LN): 0, 44, 66, 88, 90, and 100 kg/ha; middle N (MN): 107, 110, 120, 132, 150, and 153 kg/ha; and high N (HN): 176, 180, 201, 210, 335, and 480 kg/ha]. The 11 levels of irrigation (X_2_) ranged from 0 - 148.1 mm [low water (LW): 0, 52.78, 78, and 90.2 mm; middle water (MW): 91, 104.1, 104.7, and 119.2 mm; and high water (HW): 130, 133.6, and 148.1 mm].
